# CHAC1 Mediates Endoplasmic Reticulum Stress‐Dependent Ferroptosis in Calcium Oxalate Kidney Stone Formation

**DOI:** 10.1002/advs.202403992

**Published:** 2025-01-21

**Authors:** Caitao Dong, Ziqi He, Wenbiao Liao, Qinhong Jiang, Chao Song, Qianlin Song, Xiaozhe Su, Yunhe Xiong, Yunhan Wang, Lingchao Meng, Sixing Yang

**Affiliations:** ^1^ Department of Urology Renmin Hospital of Wuhan University Wuhan Hubei Province 430060 P. R. China

**Keywords:** ATF4, CHAC1, endoplasmic reticulum stress, ferroptosis, kidney stone

## Abstract

The initiation of calcium oxalate (CaOx) kidney stone formation is highly likely to stem from injury to the renal tubular epithelial cells (RTECs) induced by stimulation from an aberrant urinary environment. CHAC1 plays a critical role in stress response mechanisms by regulating glutathione metabolism. Endoplasmic reticulum (ER) stress and ferroptosis are demonstrated to be involved in stone formation. This study attempted to elucidate the mechanism of ER stress‐dependent ferroptosis and the role of CHAC1 in CaOx kidney stones. Here, regulating ER stress and CHAC1 expression are performed in in vivo and in vitro stone models. These findings indicated that 4‐Phenylbutyric acid (4‐PBA)treatment and CHAC1 deficiency alleviated the ferroptotic status, including restoring GSH content, suppressing Fe^2+^ and lipid peroxidation accumulation, as well as regulating ferroptosis‐related proteins. Notably, 4‐PBA treatment and CHAC1 deficiency both attenuated oxidative damage, improved renal function, importantly, decreased crystal deposition. Additionally, ChIP‐seq and ChIP‐qPCR analyses demonstrated that CHAC1 is the vital downstream target gene of ATF4. The results indicated that ATF4 depletion inhibited the upregulation of CHAC1 and pro‐ferroptotic response induced by Ox stimulation. Overall, ATF4/CHAC1 axis mediating ER stress‐dependent ferroptosis may be a promising research direction for identifying potential strategy to prevent and treat CaOx kidney stones.

## Graphical abstract

The molecular mechanism of ER stress‐dependent ferroptosis in CaOx stone formation is caused by the activation of ATF4/CHAC1 axis, and then the specific degradation of GSH by CHAC1 disrupts the production and biological activity of GPX4, which ultimately leads to ferroptosis. And CHAC1 deficiency can simultaneously suppress apoptotic and ferroptotic pathways to better hinder CaOx kidney stone formation.

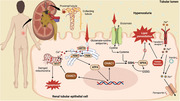

## 1 Introduction

Kidney stone is one of the most common urological diseases, with prevalence ranging from 1 to 5% in Asia, 5–9% in Europe, and 7–13% in North America.^[^ ^1^ ^]^ With the character of high incidence, high recurrence rates and high costs, kidney stone significantly impacts the quality of life and brings a considerable burden for the public and society.^[^ ^2^, ^3^ ^]^ Of those, calcium oxalate (CaOx) is the most predominant components in approximately 80% of kidney stones.^[^ ^4^, ^5^ ^]^ Although the formation of CaOx kidney stone is influenced by environmental, metabolic, genetic and other factors, numerous studies have demonstrated that the injury of renal tubular epithelial cells (RTECs) induced by abnormal urine, such as hyperoxaluria, is an important link in stone formation.^[^ ^6^, ^7^, ^8^ ^]^ Therefore, it is of great significance to explore the specific mechanism of injury and potential therapeutic targets in the formation of CaOx kidney stone.

Under normal physiological conditions, moderate endoplasmic reticulum (ER) stress response is an important mechanism for maintaining cellular homeostasis that can activate unfolded protein response (UPR) to maintain normal ER function and protein homeostasis.^[^ ^9^ ^]^ Emerging studies have shown that excessive activation of ER stress is closely associated with the development of CaOx kidney stone. When continuously exposed to oxalate or CaOx, URP was excessively activated in RTECs, resulting in injury.^[^ ^10^, ^11^, ^12^ ^]^ As a novel type of regulatory cell death, ferroptosis has also been found to be involved in stone formation. In RTECs exposed to CaOx, we observed the damaged mitochondrion, the accumulation of Fe^2+^ and increased level of lipid peroxidation. And in ethylene glycol (EG)‐induced rat stone model, inhibiting ferroptosis significantly reduced crystal aggregation.^[^ ^13^, ^14^ ^]^ It is widely demonstrated that oxidative stress plays a decisive role in the injury of RTECs induced by oxalate or CaOx,^[^ ^7^ ^]^ and the continuous oxidative stress state is an important inducement of ER stress and ferroptosis,^[^ ^10^, ^15^ ^]^ which suggests that ER stress and ferroptosis may occur together in development of CaOx kidney stone.

Interestingly, glutathione‐specific gamma‐glutamylcyclotransferase1 (CHAC1), a key enzyme that specifically catalyzes the degradation of glutathione (GSH) into a Cys‐Gly dipeptide and 5‐oxo‐L‐proline, plays a role in GSH degradation.^[^ ^16^ ^]^ Mungrue et al.^[^ ^17^ ^]^ reported that CHAC1, acting as the downstream of the UPR, especially ATF4‐CHOP pathway, participated in cell apoptosis induced by ER stress. Additionally, CHAC1 is also regarded as a ferroptosis biomarker.^[^ ^18^, ^19^ ^]^ Glutathione peroxidase 4 (GPX4) is an important detoxifying substance of intracellular lipid peroxidation toxicity, and its deficiency can induce intracellular lipid peroxidation and then trigger ferroptosis.^[^ ^20^ ^]^ Most importantly, CHAC1 can degrade GSH, serving for GPX4 as an indispensable substrate, thus weakening the activity of GPX4, leading to ferroptosis.^[^ ^21^ ^]^ However, the role and status of CHAC1 in development of CaOx kidney stone have yet to be reported.

In the present study, both in vitro and in vivo were performed to elucidate the possible pathway for ER stress‐mediated ferroptosis, and to investigate whether CHAC1 participated in modulating ER stress‐induced ferroptosis in mechanism of CaOx kidney stone formation. It is assumed that the inhibition of CHAC1 can reduce the pro‐apoptotic effect and ferroptosis induced by ER stress at the same time, which will greatly promote the basic and clinical research on the prevention and treatment of kidney stones.

## 2 Results

### 2.1 ER Stress Participated in Mediating Oxalate‐Induced Oxidative Injury and Ferroptosis in RTECs

**Figure 1:**
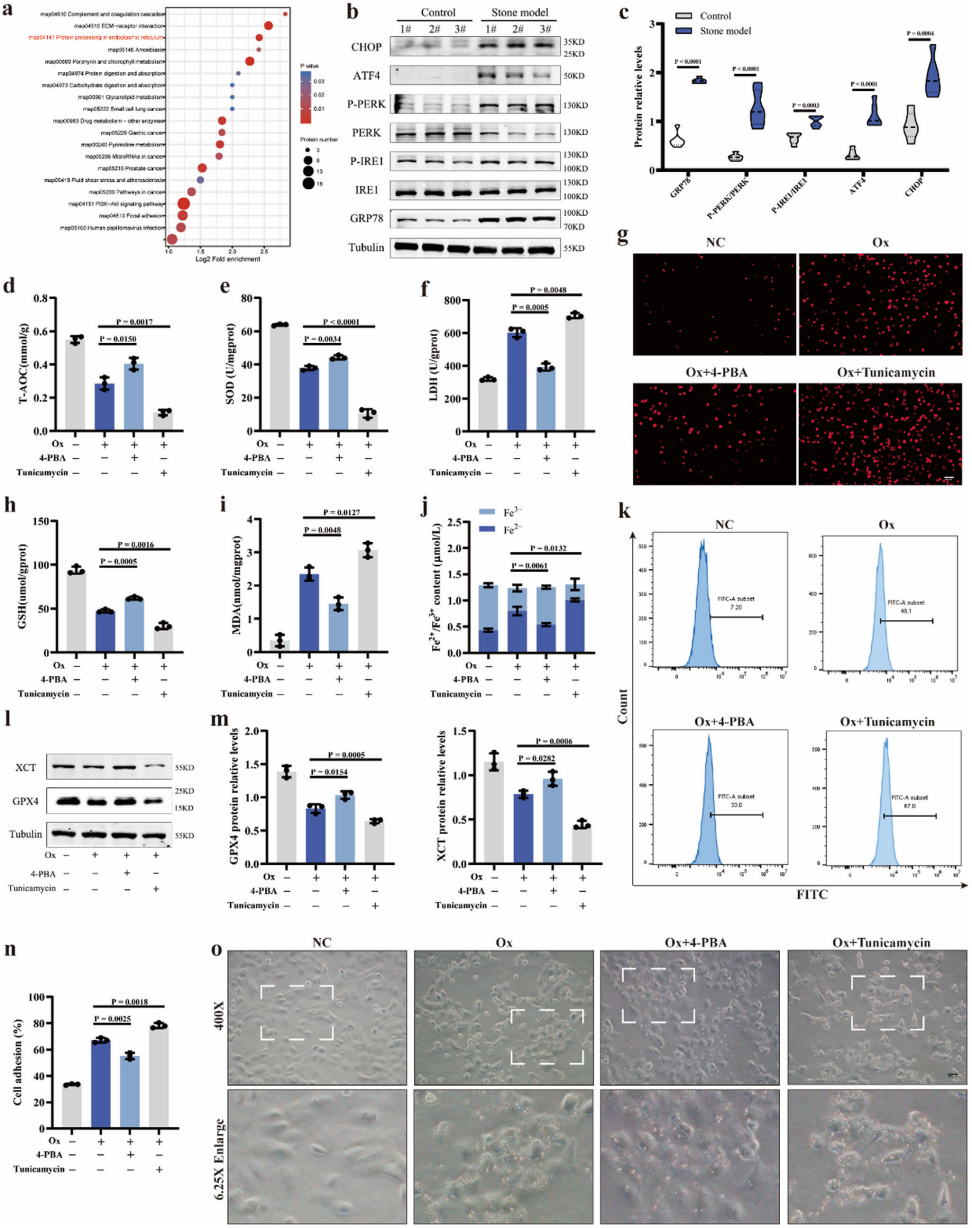
ER stress participated in mediating oxalate‐induced oxidative injury and ferroptosis in RTECs. a) Bubble plot for KEGG pathway analysis of DEGs. b) Western blotting results showed the protein expression levels of GRP78, IRE‐1, P‐IRE‐1, PERK, P‐PERK, ATF4, and CHOP for stone model group and control group. c) The violin plots show the protein relative expression according to the western blotting results. The content of T‐AOC d), SOD e), and LDH f) were detected in four groups, respectively. g) Images show the levels of cellular ROS and brighter red indicates higher levels of cellular ROS (magnification, ×200). The cellular GSH content h) and MDA content i) were measured. j) The iron assay kit was used to quantitatively detect the cellular Fe^2+^ content. k) The Liperfluo assay was used to detect the cellar degree of lipid peroxidation in four groups with flow cytometric analysis. l) Western blotting results show the protein expression levels of GPX4 and XCT for four groups and m) bar graphs show the protein relative expression. n) The ability of cell adhesion was detected. o) Images show the status of cell‐crystal adhesion between Ox crystals and HK‐2 cells (magnification, ×400). One set of representative images of three independent experiments is shown. Based on three independent experiments, data are presented as means ± SEM. P value is directly displayed and P value < 0.01 is considered significant.

First, we used glyoxylic acid to establish the mouse CaOx kidney stone models. Then, the CCK8 experiment were performed to choose an appropriate oxalate‐induced cell injury model, which suggested that 1.5 mmol L^−1^ oxalate intervening for 24 h had a moderate killing effect on HK‐2 cells (Figure S1a,b, Supporting Information). In order to search for important clues to the mechanisms of stone formation, the 4D‐LFQ quantitative proteomic analysis method was performed to elucidate the key features of proteins between oxalate‐induced HK‐2 cell injury model and normal cells (Figure S1c, Supporting Information). Through GO annotation and KEGG pathway enrichment analysis with the 4D‐LFQ quantitative proteomic data, we found that some of the differential expression proteins (DEPs) with high recommendation scores were located in the endoplasmic reticulum, and the related pathways were enriched in ER stress‐related signaling pathways (**Figure** **1**a; Figure S1d, Supporting Information). And the ER stress‐related protein expression levels of GRP78, ATF4 and CHOP, and the ratio of P‐PERK/PERK and P‐IRE1/IRE1, were significantly increased in kidney stone mouse model (Figure 1b,c). Besides, our previous study showed that the occurrence of ferroptosis is a crucial mechanism involving in CaOx kidney stone formation.^[^ ^22^ ^]^ And our results suggested that in mouse kidneys of stone models, the ferroptosis‐related proteins, including GPX4, XCT, and FTH1, were significantly decreased, while the protein expression abundance of ACSL4, CD71, and FPN1 were increased (Figure S1e,f, Supporting Information). Then, the transcript levels of ER stress‐related and ferroptosis‐related proteins were measured. We found that the GRP78, PERK, IRE1, ATF4, CHOP, and TFRC transcript levels were all significantly upregulated along with increasing concentrations of oxalate, but the transcript levels of ATF6, ACSL4 and GPX4 were not significantly altered (Figure S1g, Supporting Information).

Next, we used 4‐phenylbutyric acid (4‐PBA, ER stress inhibitor) and Tunicamycin (Tun, ER stress promotor) to modulate the degree of ER stress. To determine the effects of 4‐PBA and Tun on cell viability in our current study, the HK‐2 cells were treated with varying concentrations of 4‐PBA (0, 0.5, 1, 2.5, 5, and 10 µm) and Tunicamycin (0, 1, 2.5, 5, 10 and 20 µg mL^−1^) for 24 h. We selected 5 µM 4‐PBA (Figure S1h,i, Supporting Information) and 5 µg mL^−1^ Tun for further experiment (Figure S1j,k, Supporting Information). First, we evaluated several indicators of anti‐oxidative and oxidative status, including T‐AOC, SOD, and LDH. After 4‐PBA treatment, the Ox+4‐PBA group showed elevated T‐AOC and SOD level, and reduced LDH level than the Ox group. The levels of T‐AOC and SOD were further decreased in the Ox+Tun group compared to the Ox group, while LDH level was higher (Figure 1d–f). Then, we used DCFH‐DA to observe the ROS production of four groups, respectively. Compared to the Ox group, as shown in Figure 1g and Figure S1l (Supporting Information), the ROS production further increased after Tunicamycin treatment, but partially decreased with 4‐PBA treatment. In order to identify whether ER stress could be involved in mediating ferroptosis in oxalate‐induced RTEC injury, we detected well‐established biomarkers for ferroptosis, including MDA, the cellular content of Fe^2+^, lipid peroxidation, and key ferroptosis‐related proteins. In our present study, the GSH level was significantly recovered in the Ox+4‐PBA group but was further decreased in the Ox+Tun group compared to the Ox group (Figure 1h). The 4‐PBA treatment strikingly alleviated MDA (Figure 1i) and cellular Fe^2+^ level (Figure 1j), but the Tun intervention aggravated them in comparison to the Ox group. Subsequently, we used the Liperfluo assay to evaluate the degree of lipid peroxidation. As illustrated in Figure 1k and Figure S1m (Supporting Information), compared to the Ox group, the Tun treatment significantly worsened lipid peroxidation, which was relieved by the 4‐PBA treatment. Then, our western blotting results revealed that the protein expression of GPX4 and XCT were partially reversed after the 4‐PBA treatment and further decreased with Tunicamycin intervention (Figure 1l,m). Importantly, the ability of cell adhesion was relieved with 4‐PBA treatment and worsen with Tun intervention (Figure 1n). At the same time, by visually examining the adhesion status between Ox crystals and HK‐2 cells, we found that 4‐PBA treatment could attenuated the adhesion status, while Tun intervention aggravated it (Figure 1o; Figure S1n, Supporting Information).

### 2.2 The Positive Role of Regulating ER Stress in Inhibiting CaOx Stone Formation

**Figure 2:**
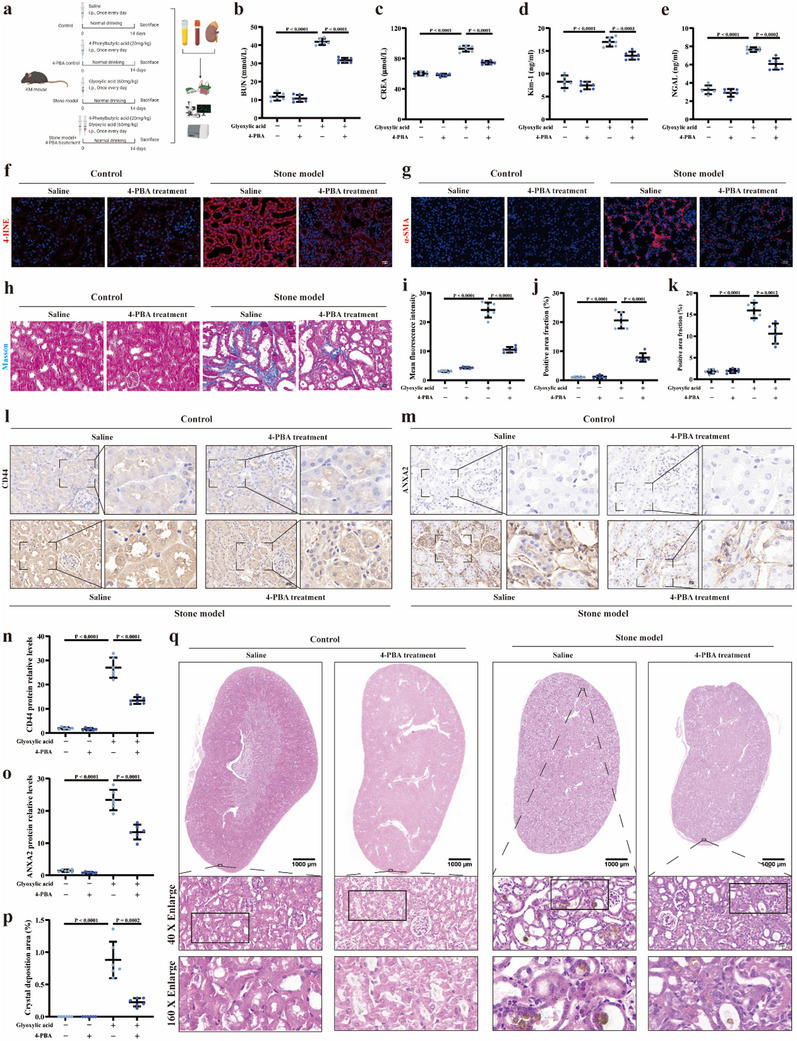
The positive role of regulating ER stress in inhibiting CaOx stone formation. a) A flowchart of the animal experiments. The bar graphs show the content of BUN b) and the content of CREA c) in blood for four groups, respectively. The Kim‐1 d) and NGAL content e) were measured using Elisa kits. f) Images show the 4‐HNE expression abundance in renal tissue for four groups (magnification, ×400). g) Images show the positive area of highly α‐SMA expression (magnification, ×400). h) Images show the degree of renal fibrosis using the Masson staining (magnification, ×400). i) Bar graph shows the mean red fluorescence intensity of 4‐HNE expression. j) Bar graph shows the positive area fraction of highly α‐SMA expression. k) Bar graph shows the positive area fraction of Masson staining. Immunohistochemical staining was performed to measure the expression abundance of CD44 l) (magnification, ×400) and ANXA2 m) (magnification, ×400). n) Bar graph shows the positive area fraction of CD44 protein expression. o) Bar graph shows the positive area fraction of ANXA2 protein expression. p) Bar graph shows the positive area fraction of crystal deposition. q) Von Kossa staining was used to display the status of crystal deposition in renal tissue (magnification, ×10 and ×400). Animal experiments were based on six independent experiments. One set of representative images of six independent experiments is shown. All data are presented as means ± SEM. P value is directly displayed and P value < 0.01 is considered significant.

As already mentioned before, ER stress and ferroptosis occurred in process of stone formation at the same time. In order to verify the beneficial effect of regulating ER stress in inhibiting ferroptosis and hindering CaOx stone formation, 20 mg kg^−1^ dose of 4‐PBA was administered to regulate the level of ER stress in CaOx stone mice models (**Figure** **2**a). First, the level of serum BUN and CRE in the 4‐PBA control group indicated that 4‐PBA treatment alone did not cause renal impairment, but 4‐PBA treatment relieved the elevated levels of serum BUN and CRE due to glyoxylic acid administration (Figure 2b,c). Then, the renal injury‐related indicators, including Kim‐1 and NGAL, were measured in four groups, and our results showed that the renal expression of Kim‐1 and NGAL were significantly decreased in the 4‐PBA treatment group compared to the stone model group (Figure 2d,e). And 4‐PBA treatment also reduced 4‐HNE production in renal tissue of mice with kidney stones (Figure 2f,i). Furthermore, as shown in Figure 2g,h, the positive role of regulating ER stress in alleviating renal fibrosis in mouse kidney stone models was visually evaluated according to α‐SMA and Masson staining. The immunofluorescence analysis demonstrated that 4‐PBA treatment greatly ameliorated the degree of renal fibrosis observed in the stone model group (Figure 2j,k). Furthermore, the western blotting results demonstrated that in 4‐PBA treatment group, the expression of crystal adhesion‐associated proteins, including CD44 and ANXA2, were downregulated, at the same time (Figure 2l–o). And we also observed that, compared to the stone model group, in 4‐PBA treatment group, the crystal deposition was dramatically decreased (Figure 2p,q). Our above results demonstrated that regulating overwhelmed ER stress could positively hinder CaOx stone formation by alleviating renal impairment.

### 2.3 ER Stress‐Associated ATF4/CHAC1 Axis was Activated in CaOx Kidney Stone Model

**Figure 3:**
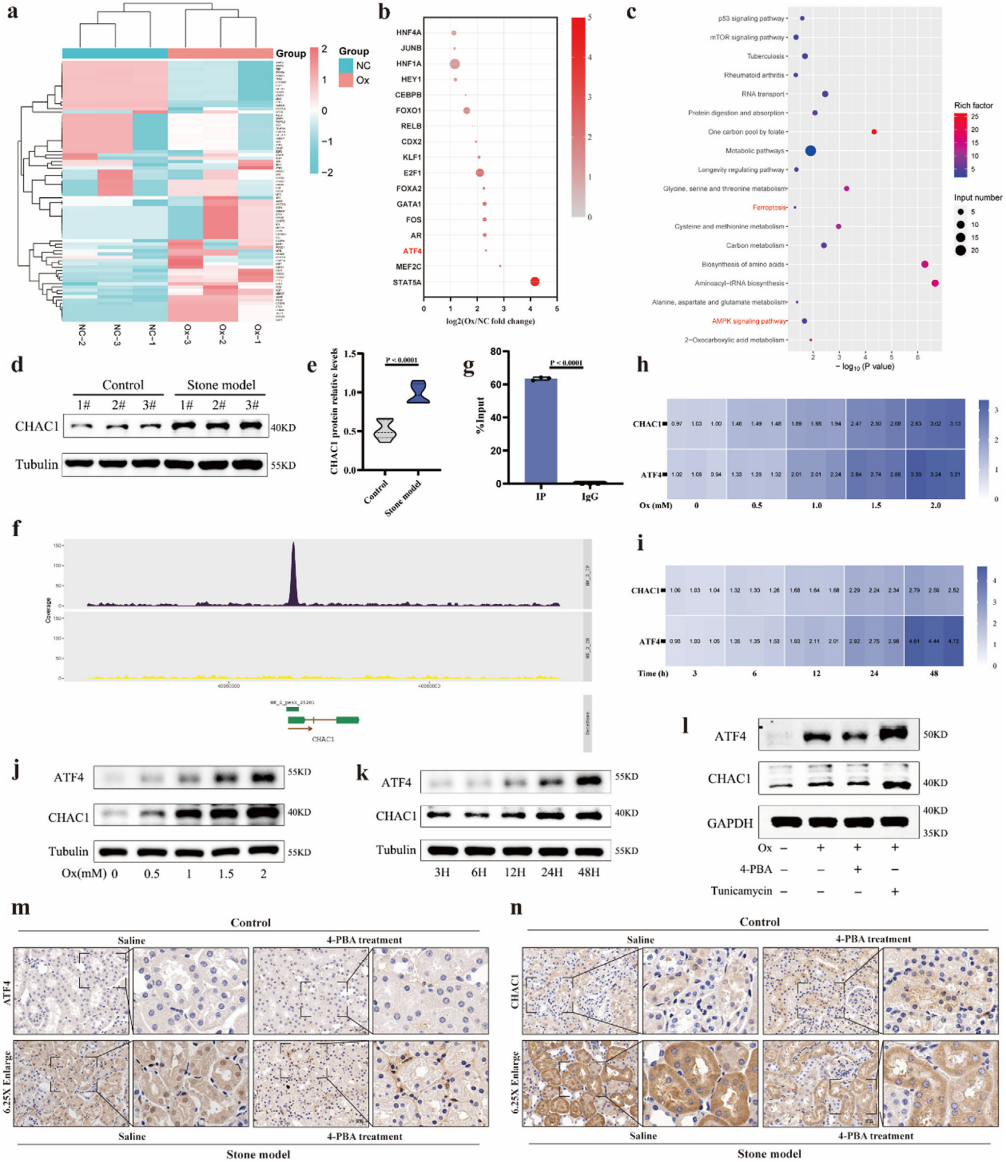
ER stress‐associated ATF4/CHAC1 axis was activated in CaOx kidney stone model. a) The heatmap plot shows differential expression changes of all transcription factors in the human transcription factors PCR Array plate. b) The bubble plot shows differential expression changes of all transcription factors related to ER stress. The size of bubbles represents the amount of P value. The color of bubbles represents the amount of fold change. c) Bubble plot of KEGG in ChIP‐seq. d) Western blotting results show the protein expression levels of CHAC1 for stone model group and control group and e) bar graph shows the protein relative expression. f) ChIP‐seq verified the distribution of ATF4 reads over the CHAC1 gene. g) ChIP‐qPCR verified the transcription of CHAC1 by ATF4 targeting. h) qPCR results show the transcriptional levels of ATF4 and CHAC1 followed by Ox concentration gradient incubating for 24 h. i) qPCR results show the transcriptional levels of ATF4 and CHAC1 followed by time gradient with 1.5 mM Ox incubation. j) Western blotting results show the protein expression levels of ATF4 and CHAC1 followed by Ox concentration gradient incubating for 24 h. k) Western blotting results show the protein expression levels of ATF4 and CHAC1 followed by time gradient with 1.5 mm Ox incubation. l) Western blotting results show the protein expression levels of ATF4 and CHAC1 for four groups. m) Immunohistochemical staining was performed to measure the expression abundance of ATF4 protein (magnification, ×400). n) Immunohistochemical staining was performed to measure the expression abundance of CHAC1 protein (magnification, ×400). One set of representative images is shown. In vitro experiments were based on three independent experiments and in vivo experiments were based on six independent experiments, data are presented as means ± SEM. P value is directly displayed and P value < 0.01 is considered significant.

Subsequently, in order to explore the exact mechanism of ER stress‐modulated injury, we used the human transcription factors PCR Array plate to screen key transcriptional regulators (**Figure** **3**a). The transcription factor Array analysis showed that the alteration of ATF4 transcription level had excellent difference and significance between oxalate‐induced HK‐2 cell injury model and normal cells (Figure 3b). Based on the screened KEGG pathway, protein processing in endoplasmic reticulum, in the 4D‐LFQ quantitative proteomic data (Figure S2a, Supporting Information), we had demonstrated that the PERK/ATF4 pathway, an important branch of UPR, was apparently activated in CaOx kidney stone model (Figure 1b). Furthermore, we conducted the ChIP‐seq experiment to search for downstream target genes of ATF4 (Figure S2b,c, Supporting Information). Then, KEGG pathway annotation analysis of target genes indicated several related pathways, including metabolic pathways, ferroptosis, AMPK signaling pathway, and so on (Figure 3c). Through GO annotation analysis of target genes, all target genes associated with UPR were selected (Figure S2d, Supporting Information) and CHAC1 gene has the highest fold enrichment (Figure S2e, Supporting Information). And in kidney stone mouse model, renal expression of CHAC1 was apparently increased (Figure 3d,e). The distribution of ATF4 reads over the CHAC1 gene is shown in Figure 3f. Taking a further step, ChIP‐qPCR analysis was conducted to validate the association between ATF4 and CHAC1 gene, and our results demonstrated that ATF4 protein has a significant interaction relationship with downstream CHAC1 gene (Figure 3g).

Then, qPCR results indicated that both ATF4 and CHAC1 mRNA were upregulated with the increasing Ox concentration and incubation time gradient (Figure 3h,i). Similarly, both ATF4 and CHAC1 protein expression were also progressively increased, wherein, the 1.5 mm Ox treatment for 24 h were used for further research (Figure 3j,k; Figure S2f,g, Supporting Information). To continue investigating whether ER stress mediated CHAC1 in CaOx kidney stone formation, the 4‐PBA and Tunicamycin were performed again. Compared to the Ox group, the Ox+4‐PBA group showed downregulated ATF4 and CHAC1 protein expression level with 4‐PBA treatment, while the protein level of ATF4 and CHAC1 were severely increased in the Ox+Tun group at the same time (Figure 3l; Figure S2h–j, Supporting Information). Furthermore, in vivo, the immunohistochemistry results showed that 4‐PBA treatment greatly reduced both ATF4 and CHAC1 expression in the stone model+4‐PBA treatment group compared to stone model group (Figure 3m,n; Figure S2i–k, Supporting Information).

### 2.4 The ATF4/CHAC1/GPX4 Pathway was Involved in ER Stress‐Dependent Ferroptosis in CaOx Kidney Stone Model

**Figure 4:**
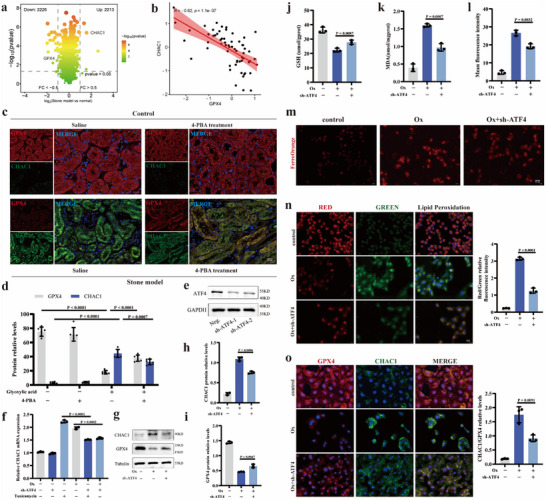
The ATF4/CHAC1/GPX4 pathway was involved in ER stress‐dependent ferroptosis in CaOx kidney stone model. a) Volcano plot of DEGs in 4D‐LFQ quantitative proteomic analysis. b) The linearity graph shows the association between CHAC1 and GPX4 in nephrolithiasis patients based on the nephrolithiasis gene expression datasets (GSE73680). c) Immunofluorescence staining was performed to simultaneously detect the CHAC1 and GPX4 protein expression abundance in renal tissue (magnification, ×400) and d) bar graph shows the protein relative expression. e) The validation of ATF4 knockdown by western blot. f) The CHAC1 mRNA expression in HK‐2 cells were determined using qRT‐PCR analysis for five groups. g) Western blotting results show the protein expression levels of CHAC1 and GPX4 for four groups and h,i) bar graphs show the protein relative expression. The GSH j) content and MDA k) content were detected in three groups. l) The FerroOrange assay was used to qualitatively detect the cellular Fe^2+^ content. Bar graph shows the mean red fluorescence intensity and m) images were taken under a dark field (magnification, ×400). Brighter red indicates higher cellular Fe^2+^ content. n) The BDP 581/591 C11 assay was used to qualitatively measure the degree of lipid peroxidation and bar graph shows the ratio of red to green fluorescence (magnification, ×400). The lower the ratio, the worse the degree of lipid peroxidation. o) Immunofluorescence staining was performed to simultaneously detect the cellular CHAC1 and GPX4 protein expression abundance (magnification, ×400) and bar graph shows the ratio of CHAC1 expression to GPX4 expression. One set of representative images of three independent experiments is shown. Based on three independent experiments, data are presented as means ± SEM. P value is directly displayed and P value < 0.01 is considered significant.

CHAC1 is involved in GSH degradation.^[^ ^18^, ^19^ ^]^ GPX4 is a critical mediator in inhibiting lipid peroxidation and ferroptosis, and GSH serves as a cofactor for GPX4 activity.^[^ ^20^ ^]^ Notably, the GSH level was significantly depleted in both in vivo and in vitro stone model (Figure 1h and 8a). We found that GPX4 and CHAC1 were both DFPs with high recommendation scores in 4D‐LFQ quantitative proteomic analysis (**Figure** **4**a). And we further used the nephrolithiasis gene expression datasets (GSE73680), which found a strong negative association between GPX4 and CHAC1 (Figure 4b). We simultaneously detected CHAC1 and GPX4 protein expression in the renal using immunohistochemistry analysis. Our results indicated that the GPX4 protein expression was partially increased in the 4‐PBA treatment group compared to the stone model group, and 4‐PBA administration also significantly attenuated the elevated CHAC1 protein expression induced by glyoxylic acid (Figure 4c,d). Then, we manipulated ER stress pathway by knocking down ATF4 with LV vectors containing sh‐ATF4, which was validated by western blotting (Figure 4e). We detected the CHAC1 mRNA level by qPCR in HK‐2 cells with Tun or Ox intervention after ATF4 knockdown. Our results showed that the knockdown of ATF4 significantly reduced the effect of Tun or Ox intervention on stimulating the transcription of CHAC1 (Figure 4f). Then, as shown in Figure 4g–i, the knockdown of ATF4 potently reversed CHAC1 upregulation and GPX4 depletion induced by Ox intervention. Similarly, the GSH depletion (Figure 4j) and MDA accumulation (Figure 4k) induced by Ox intervention was effectively alleviated due to the knockdown of ATF4. And ATF4 knockdown also relieved the accumulation of Fe^2+^ (Figure 4l,m) and lipid peroxides (Figure 4n) compared to Ox group. Additionally, the abundant of CHAC1 and GPX4 was simultaneously detected using the immunofluorescence assay, which showed that ATF4 deficiency indeed attenuated oxalate‐induced the upregulation of CHAC1, as well as the downregulation of GPX4 (Figure 4o).

### 2.5 CHAC1 Mediated Oxalate‐Induced Oxidative Injury and Apoptosis in RTECs

**Figure 5:**
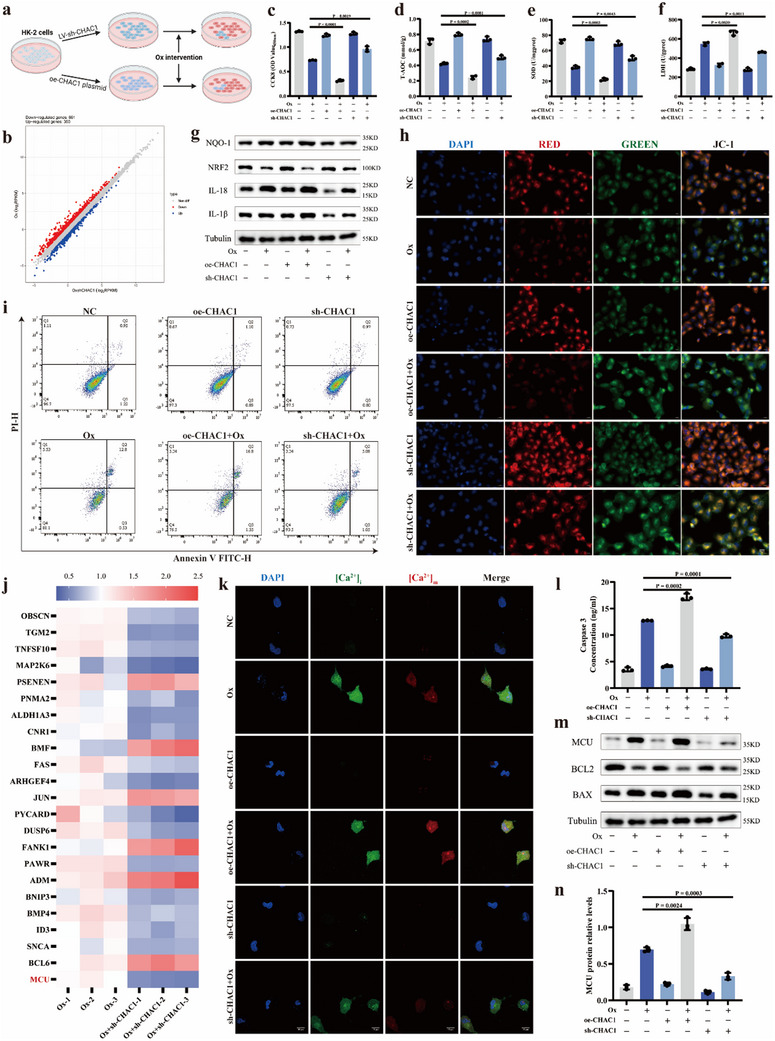
CHAC1 mediated oxalate‐induced oxidative injury and apoptosis in RTECs. a) LV vector for CHAC1 knockdown and the plasmid for CHAC1 overexpression were used to construct sh‐CHAC1 and oe‐CHAC1 HK‐2 cell lines. b) Scatter plot for DEPs in RNA‐seq experiment. c) The cell viability for six groups was detected using CCK‐8 assay. The T‐AOC content d), SOD content e), and LDH content f) was measured in each group. g) Western blotting results show the protein expression levels of NQO‐1, NRF2, IL‐18, and IL‐1β for six groups. h) The JC‐1 assay was performed to determine the mitochondrial membrane potential of HK‐2 cells (magnification, ×400). The lower the ratio of red to green fluorescence, the worse the mitochondrial membrane potential. i) The flow cytometry was used detect the apoptotic condition for six groups. j) The heatmap shows differential expression changes of all DEPs related to apoptotic pathways. k) The Fluo‐4, AM and Rhod‐2, AM were used to detect intracellular Ca^2+^ content and mitochondrial Ca^2+^ content, respectively (magnification, ×1000). l) The cellular caspase 3 activity for six groups was detected. m) Western blotting results show the protein expression levels of BAX, BCL2 and MCU for six groups. n) Bar graphs show the MCU protein relative expression for six groups. One set of representative images of three independent experiments is shown. Based on three independent experiments, data are presented as means ± SEM. P value is directly displayed and P value < 0.01 is considered significant.

To further explore the role of CHAC1 protein in mechanism of CaOx kidney stone formation, LV vector for CHAC1 knockdown and the plasmid for CHAC1 overexpression were used to construct sh‐CHAC1 and oe‐CHAC1 HK‐2 cell lines (**Figure** **5**a), respectively, which were further validated by the western blotting experiment (Figure S3a,b, Supporting Information). Then, after CHAC1 knockdown, we exerted the RNA‐seq analysis to investigate CHAC1‐mediated downstream mechanism (Figure S3c, Supporting Information). In comparison to the Ox group, the Ox+sh‐CHAC1 group exhibited 1011 DEPs, including 661 downregulated proteins and 350 upregulated proteins (Figure 5b). GO enrichment analyses illustrated that the DEGs were enriched in 226 GO terms. Of these, the term, referred to positive regulation of inflammatory response, was highlighted, which suggested that CHAC1 may mediate oxalate‐induced inflammatory response and oxidative injury in RTECs (Figure S3d, Supporting Information). First, the CHAC1 deficiency partially alleviated the cell viability of oxalate‐induced HK‐2 cells, while CHAC1 overexpression exacerbated the loss of cell viability (Figure 5c). After the knockdown of CHAC1, the Ox+sh‐CHAC1 group showed elevated T‐AOC and SOD level, and reduced LDH level than the Ox group. The levels of T‐AOC and SOD were further decreased in the Ox+oe‐CHAC1 group compared to the Ox group, while LDH level was higher (Figure 5d–f). Then, we measured the protein levels of inflammatory and oxidative stress response proteins, including IL‐1β, IL‐18, Nrf2, and NQO‐1, and found that Ox intervention apparently increased the protein expression of IL‐1β, IL‐18, and NQO‐1, and decreased the Nrf2 protein abundance. Then, the knockdown of CHAC1 apparently alleviated this change, while CHAC1 overexpression exacerbated it (Figure 5g; Figure S3e, Supporting Information).

The occurrence of apoptosis in RTECs has been largely demonstrated to be a vital mechanism in formation process of CaOx kidney stones.^[^ ^7^, ^23^ ^]^ Of note, it was reported that CHAC1 participates in triggering apoptosis.^[^ ^24^, ^25^ ^]^ We used JC‐1 assays to observe the mitochondrial function of six groups, respectively. Compared to the Ox group, as shown in Figure 5h and Figure S3f (Supporting Information), the mitochondrial membrane potential further increased after CHAC1 overexpression, but partially decreased with CHAC1 deficiency. Then, the flow cytometry was performed to detect the early and late apoptotic rate for six groups. Our results revealed that Ox dramatically increased HK‐2 cell apoptosis, a high early and late apoptotic rate. At the same time, the knockdown of CHAC1 apparently alleviated oxalate‐triggered apoptosis, while CHAC1 overexpression exacerbated it (Figure 5i; Figure S3g, Supporting Information). Furthermore, we screened all apoptosis‐related genes with high recommendation score in RNA‐sequence data. Of these, MCU, mitochondrial calcium uniporter, was most obviously downregulated after CHAC1 knockdown (Figure 5j). Notably, endoplasmic reticulum and mitochondria play critical roles as substantial intracellular calcium ion reservoirs, significantly influencing calcium ion balance in the cytoplasm. And the imbalance of calcium ion concentration would induce apoptosis.^[^ ^26^ ^]^ Then, we measured the concentration of intracellular and mitochondrial calcium ions. The level of intracellular calcium ions appeared to be slightly fluctuated in Ox+oe‐CHAC1 group and Ox+sh‐CHAC1 group compared to the Ox group. And CHAC1 overexpression caused more mitochondrial calcium accumulation in Ox+oe‐CHAC1 group, while the knockdown of CHAC1 significantly alleviated mitochondrial calcium overload in Ox+sh‐CHAC1 group (Figure 5k; Figure S3h,i, Supporting Information). At the same time, compared to the Ox group, the levels of Caspase 3 activity were further increased along with mitochondrial calcium overload in the Ox+oe‐CHAC1 group, and were significantly decreased in the Ox+sh‐CHAC1 group (Figure 5l). And the western blotting results also demonstrated that the MCU protein expression (Figure 5m,n) and the ratio of BAX/BCL2 (Figure 5m; Figure S3j, Supporting Information) further increased after CHAC1 overexpression, but apparently decreased with CHAC1 deficiency, compared to Ox group.

### 2.6 CHAC1 Deficiency Ameliorated the Renal Impairment and Improved Renal Function in the Mouse Kidney Stone Model

**Figure 6:**
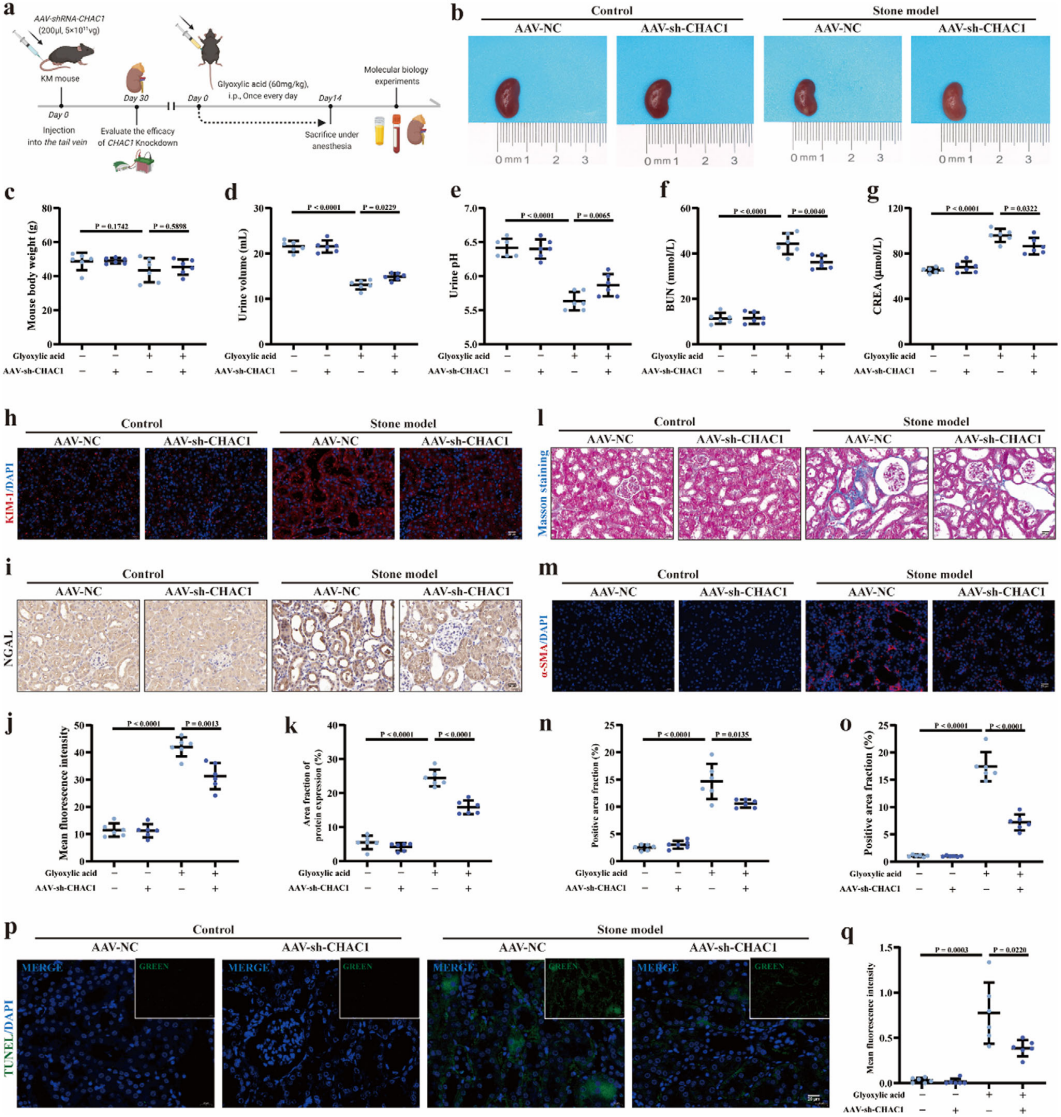
CHAC1 deficiency ameliorated the renal impairment and improved renal function in the mouse kidney stone model. a) AAV‐sh‐CHAC1 was injected 30 days in advance to inhibit CHAC1 expression in the kidneys and then glyoxylic acid was used to establish mouse kidney stone model. b) Images show the mouse kidneys from four groups, respectively. c) The bar graph shows the mouse body weight for four groups. The bar graphs show the urine volume d) and urine pH e) for four groups, respectively. The bar graphs show the content of BUN f) and the content of CREA g) in blood for four groups, respectively. h) Images show the expression abundant of Kim‐1 for four groups. i) Immunohistochemical staining was used to measure NGAL expression in renal tissue (magnification, ×400). j) Bar graph shows the mean red fluorescence intensity of Kim‐1 expression. k) Bar graph shows the positive area fraction of highly NGAL expression. l) Images show the degree of renal fibrosis using the Masson staining (magnification, ×400). m) Images show the positive area of highly α‐SMA expression (magnification, ×400). n) Bar graph shows the positive area fraction of Masson staining. o) Bar graph shows the positive area fraction of highly α‐SMA expression. p) The TUNEL staining was performed to detect the apoptotic level in renal tissue (magnification, ×400) and q) bar graph show the mean green fluorescence intensity. In vivo experiments were based on six independent experiments. One set of representative images of six independent experiments is shown. All data are presented as means ± SEM. P value is directly displayed and P value < 0.01 is considered significant.

To further validate the mechanism of CHAC1 in CaOx kidney stones, we injected AAV‐sh‐CHAC1 30 days in advance to inhibit CHAC1 expression in the kidneys before establishing mouse kidney stone models (**Figure** **6**a). As shown in Figure 6b,c, the size of kidneys and body weight in mouse with glyoxylic acid supplement and injecting AAV‐sh‐CHAC1 had no apparent alternation. For urine volume (Figure 6d) and urine PH (Figure 6e), the mouse kidney stone model both showed a marked decline, while CHAC1 deficiency alleviated the urine volume and urine PH, to a certain degree. For renal function, in the stone model group, the blood urea nitrogen (BUN) and serum creatinine (CRE) were significantly increased. And injecting AAV‐sh‐CHAC1 in normal mouse did not cause renal function impairment. While, the knockdown of CHAC1 obviously downregulated the increase of BUN and CRE induced by glyoxylic acid (Figure 6f,g). Then, the indicators of renal impairment, Kim‐1 (Figure 6h,j) and NGAL (Figure 6i,k), were measured and our results showed that CHAC1 knockdown in normal mouse also did not cause kidney injury, but in mouse kidney stone models could mildly alleviate renal impairment. And the Masson staining (Figure 6l,n) and α‐SMA (Figure 6m,o) staining demonstrated that the degree of renal fibrosis induced by CaOx crystals was diminished benefitting from CHAC1 knockdown. Additionally, we further detected the apoptotic status of renal tissue, and found that CHAC1 deficiency also mildly restored apoptotic conditions triggered by CaOx crystals (Figure 6p,q).

### 2.7 CHAC1 Modulated ER Stress‐Dependent Ferroptosis in Ox‐Induced RTECs

**Figure 7:**
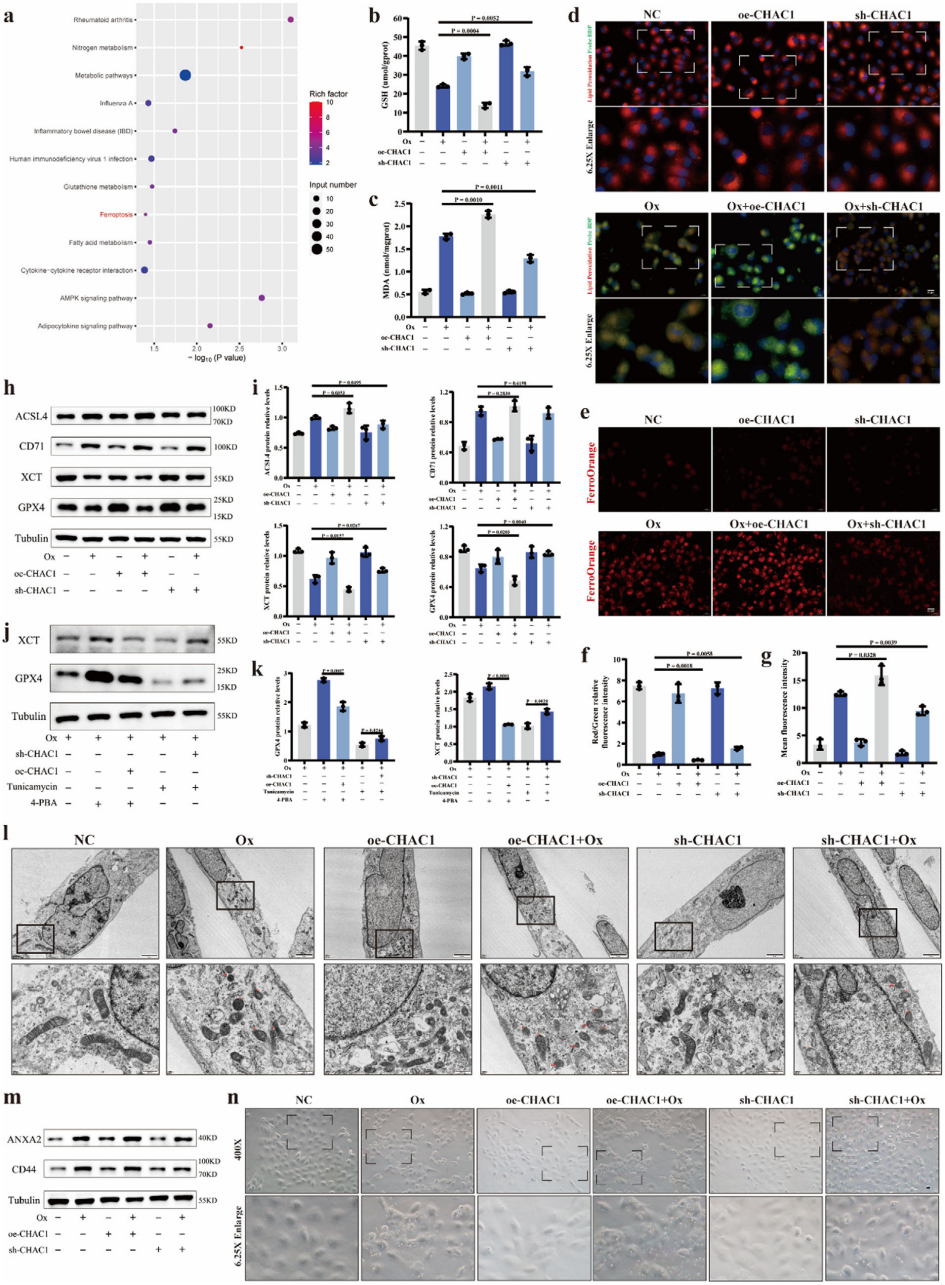
CHAC1 modulated ER stress‐dependent ferroptosis in Ox‐induced RTECs. a) Bubble plot of KEGG pathway in RNA‐seq experiment. b) The content of GSH was detected for six groups. c) The content of MDA was detected for six groups. d) The BDP 581/591 C11 assay was used to qualitatively measure the degree of lipid peroxidation (magnification, ×400). e) The FerroOrange assay was used to qualitatively detect the cellular Fe^2+^ content. Images were taken under a dark field (magnification, ×400). f) Bar graph shows the ratio of red to green fluorescence. The lower the ratio, the worse the degree of lipid peroxidation. g) Bar graph shows the mean red fluorescence intensity. Brighter red indicates higher cellular Fe^2+^ content. h) Western blotting results show the protein expression levels of GPX4, XCT, CD71, and ACSL4 for six groups and i) bar graphs show the protein relative expression. j) Western blotting results show the protein expression levels of GPX4 and XCT for five groups and k) bar graphs show the protein relative expression. l) Images show the mitochondrial morphology alterations associated with ferroptosis under transmission electron microscopy. (scale bar = 2 µm, 0.5 µm). Red asterisks indicate mitochondria with reduced mitochondrial cristae and ruptured mitochondrial membrane, and red arrows indicate shrunk mitochondria. m) Western blotting results show the protein expression levels of ANXA2 and CD44 for six groups. n) Images show the status of cell‐crystal adhesion between Ox crystals and HK‐2 cells (magnification, ×400). One set of representative images of three independent experiments is shown. Based on three independent experiments, data are presented as means ± SEM. P value is directly displayed and P value < 0.01 is considered significant.

Of concern, through KEGG pathway enrichment analysis in RNA sequence analysis, ferroptosis is recommended as a crucial downstream pathway with CHAC1 modulating (**Figure** **7**a). Thus, the ferroptosis‐related indicators were measured in sh‐CHAC1 and oe‐CHAC1 HK‐2 cell lines. Notably, the CHAC1 deficiency largely improved the content of GSH. At the same time, the CHAC1 overexpression heavily shortened the GSH level (Figure 7b). The elevated MDA level induced by Ox intervention was effectively attenuated due to the knockdown of CHAC1 but ulteriorly elevated by CHAC1 overexpression (Figure 7c). Then, the CHAC1 deficiency significantly alleviated the level of lipid peroxidation (Figure 7d,f) and Fe^2+^ (Figure 7e,g) in comparison with the Ox group, while CHAC1 overexpression further exacerbated them. Meanwhile, compared to the Ox group, the protein expression of GPX4 and XCT were obviously increased, and the ACSL4 protein expression was decreased in the Ox+sh‐CHAC1 group. In the Ox‐oe‐CHAC1 group, the GPX4 and XCT protein expression were severely reduced and the ACSL4 expression was further increased. However, CHAC1 overexpression or deficiency had a little impact on CD71 expression (Figure 7h,i). And immunofluorescence staining again validated the impact of modulating CHAC1 on GPX4 and XCT protein expression (Figure S4a–c, Supporting Information). Subsequently, the role of CHAC1 in modulating ER stress‐mediated ferroptosis in Ox‐stimulated RETCs was further investigated. The western blotting experiment results showed that CHAC1 deficiency or overexpression had no obvious effects on ER stress‐related protein expression levels of GRP78, ATF4, and the ratio of P‐PERK/PERK (Figure S4d,e, Supporting Information). Despite the 4‐PBA supplement, CHAC1 overexpression dramatically reduced the content of GPX4 and XCT protein. And the depletion of GPX4 and XCT protein with Tun intervention was apparently reversed due to the knockdown of CHAC1, to a certain degree (Figure 7j,k). And we observed mitochondrial morphology using the transmission electron microscopy. In Ox group, we found the reduction of mitochondrial cristae, the rupture of mitochondrial membrane, and shrunk mitochondria. And these Ox‐induced changes of mitochondria were alleviated in the Ox+sh‐CHAC1 group, but mitochondria were further damaged after CHAC1 overexpression (Figure 7l). Taken together, these data clearly implicated that CHAC1 was partially involved in mediating oxalate‐induced ferroptosis. Furthermore, researchers proposed that damaged RTECs may be the onset of stone formation.^[^ ^7^ ^]^ And more cell adhesion molecules, providing binding sites for crystals, would be upregulated when cell injury occurred, which further fostered cell–crystal adhesion.^[^ ^4^, ^27^ ^]^ First, the expression level of two key cell adhesion molecules, CD44 and ANXA2, were measured, which suggested that Ox intervention significantly triggered RETCs to produce a mass of CD44 and ANXA2 protein. In the Ox+sh‐CHAC1 group, the abundance of CD44 and ANXA2 protein were downregulated compared to the Ox group, while they were further upregulated in the Ox+oe‐CHAC1 group (Figure 7m; Figure S4f, Supporting Information). Then, the ability of cell adhesion (Figure S4g, Supporting Information) and status of cell‐crystal adhesion (Figure 7n; Figure S4h, Supporting Information) was observed and our results showed that CHAC1 knockdown attenuated the adhesion status between Ox crystals and HK‐2 cells, while CHAC1 overexpression aggravated it.

### 2.8 The Ferroptosis‐Apoptosis Synergistic Effects in CaOx Kidney Stone Models

In our study, we clearly recognized that ferroptosis and apoptosis were both involved in the mechanism of CaOx kidney stone formation. In order to investigate the relationship between these two in CaOx kidney stone models, Ferrostatin‐1 (Fer‐1, ferroptosis inhibitor) and Z‐VAD‐FMK (apoptosis inhibitor) were used. To determine the effects of Fer‐1 and Z‐VAD‐FMK on cell viability, the HK‐2 cells were treated with varying concentrations of Fer‐1 and Z‐VAD‐FMK for 24h. We selected 10 µm Z‐VAD‐FMAK (Figure S5a,b, Supporting Information) and 5 µm Fer‐1 for further experiment (Figure S5c,d, Supporting Information). Our results showed that the early and late apoptotic rate, the Caspase 3 activity, and the concentration of intracellular and mitochondrial calcium ions were all moderately decreased with Fer‐1 treatment (Figure S5e–j, Supporting Information). And concomitant use of Fer‐1 and Z‐VAD‐FMK further significantly reduced the apoptotic rate, the Caspase 3 activity, and intracellular and mitochondrial calcium ion level (Figure S5e–j, Supporting Information). Although overexpression of CHAC1 protein aggravated the extent of RTEC apoptosis, Fer‐1 and Z‐VAD‐FMK treatment had a beneficial effect on relieving it (Figure S5e–j, Supporting Information). Further, compared to Ox group and oe‐CHAC1+Ox, respectively, the cleaved Caspase 3 and MCU protein expression and the ratio of BAX and BCL2 protein was significantly decreased after concomitant treatment of Fer‐1 and Z‐VAD‐FMK (Figure S5n,o, Supporting Information). Similarly, as is shown in Figure S6b (Supporting Information), we further detected the apoptotic status of renal tissue, and found that Fer‐1 and Z‐VAD‐FMK treatment both alleviated apoptotic conditions triggered by CaOx crystals. And immunohistochemical results indicated that concomitant use of Fer‐1 and Z‐VAD‐FMK further decreased the upregulated MCU expression (Figure S6e, Supporting Information) observed in the stone model group.

At the same time, our results showed that Z‐VAD‐FMK alone treatment slightly alleviated the increased levels of MDA and lipid peroxidation induced by Ox intervention, and these two‐agent combination therapy further improved them (Figure S5k–m, Supporting Information). Then, compared to Ox group and oe‐CHAC1+Ox, respectively, the deficiency of GPX4 and XCT protein were significantly improved after concomitant treatment of Fer‐1 and Z‐VAD‐FMK (Figure S5n,o, Supporting Information). In vivo, Z‐VAD‐FMK alone treatment moderately reduced the level of 4‐HNE and elevated the GSH content in comparison to Stone model group, and concomitant treatment of Fer‐1 and Z‐VAD‐FMK had better ameliorative effects (Figure S6c,d, Supporting Information). Interestingly, we found that the use of Fer‐1 and Z‐VAD‐FMK had a little impact on CHAC1 protein, but apparently improved the renal GPX4 protein expression (Figure S6f,g, Supporting Information). Furthermore, our results indicated that the ability of cell adhesion (Figure S5p, Supporting Information) and status of cell‐crystal adhesion (Figure S5q, Supporting Information) were also alleviated by Fer‐1 and Z‐VAD‐FMK treatment, to some degree. And after Fer‐1 or/and Z‐VAD‐FMK treatment, the status of crystal deposition was decreased compared with the stone model group, and Fer‐1 treatment alone worked better than Z‐VAD‐FMK treatment alone (Figure S6h, Supporting Information). Besides, concomitant use of Fer‐1 and Z‐VAD‐FMK had a better favorable effect on alleviating cell‐crystal adhesion and crystal composition (Figures S5p,q and S6h, Supporting Information). These results suggested that two‐agent combination therapy better suppressed ferroptosis and apoptosis than a single agent, and simultaneously inhibiting ferroptosis and apoptosis could better hinder CaOx kidney stone formation.

### 2.9 CHAC1 Deficiency Suppressed Ferroptosis and Alleviated Crystal Deposition in the Mouse Kidney Stone Model

**Figure 8:**
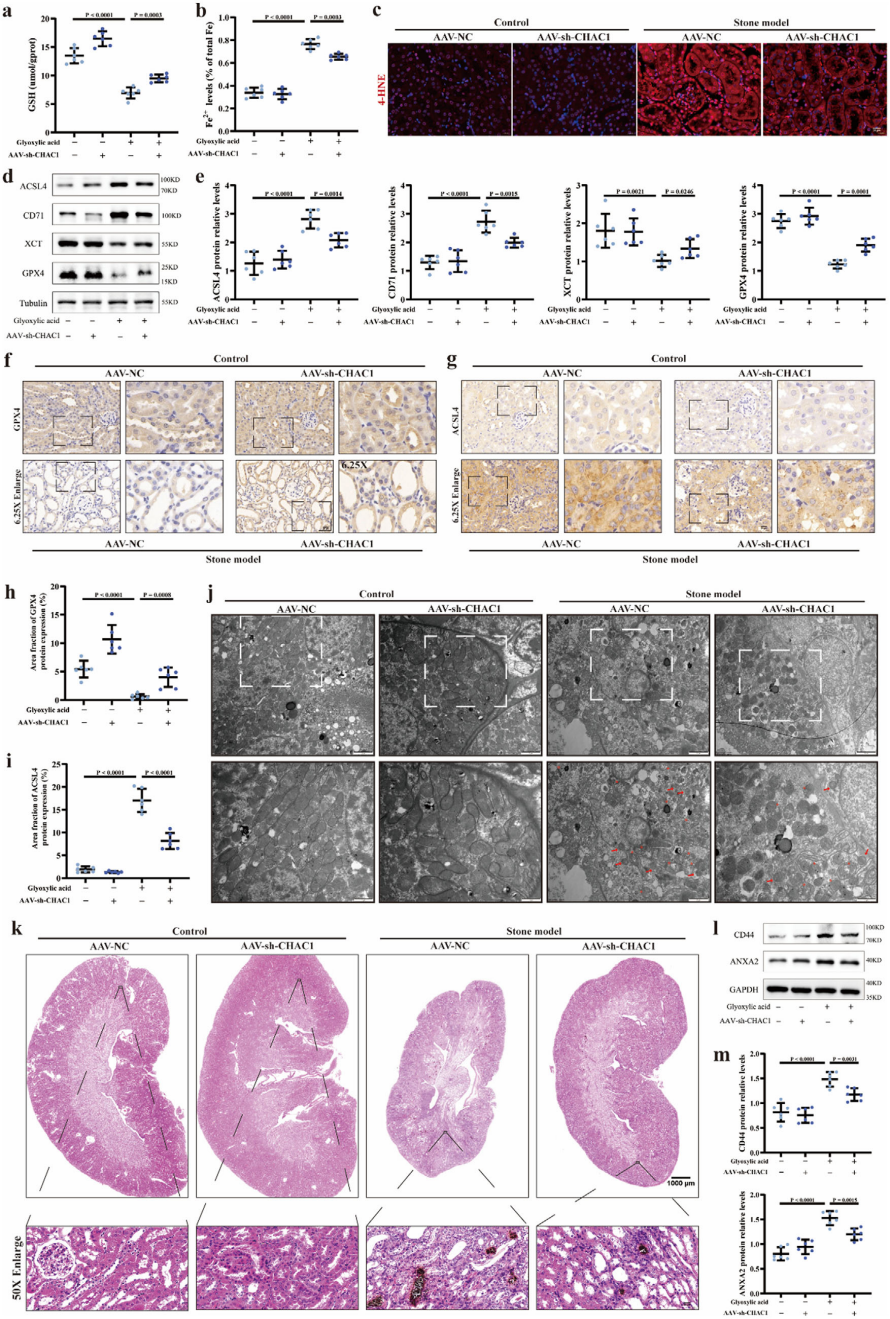
CHAC1 deficiency suppressed ferroptosis and alleviated crystal deposition in the mouse kidney stone model. The content of GSH a) and the content of Fe^2+^ b) in renal tissue were detected for four groups. c) Images show the 4‐HNE expression abundance in renal tissue for four groups (magnification, ×400). d) Western blotting results show the protein expression levels of GPX4, XCT, CD71, and ACSL4 for four groups and e) bar graphs show the protein relative expression. f) Immunohistochemical staining was used to measure GPX4 protein expression in renal tissue (magnification, ×400). g) Immunohistochemical staining was used to measure ACSL4 protein expression in renal tissue (magnification, ×400). h) Bar graph shows the positive area fraction of highly GPX4 expression. i) Bar graph shows the positive area fraction of highly ACSL4 expression. j) Images show the mitochondrial morphology alterations associated with ferroptosis under transmission electron microscopy (scale bar = 2 µm, 1 µm). Red asterisks indicate mitochondria with reduced mitochondrial cristae and ruptured mitochondrial membrane, and red arrows indicate shrunk mitochondria. k) Von Kossa staining was used to display the status of crystal deposition in renal tissue (magnification, ×8 and ×400). l) Western blotting results show the protein expression levels of CD44 and ANXA2 for four groups and m) bar graphs show the protein relative expression. Animal experiments were based on six independent experiments. One set of representative images of six independent experiments is shown. All data are presented as means ± SEM. P value is directly displayed and P value < 0.01 is considered significant.

Next, the GSH level was detected in four groups and our results revealed that CHAC1 knockdown slightly elevated the GSH level of normal mouse, and also partially reversed its decrease induced by glyoxylic acid intervention (**Figure** **8**a). Then, the efficacy of CHAC1 deficiency on modulating ferroptosis was evaluated in renal tissue. We found that the content of Fe^2+^ (Figure 8b) and the level of 4‐HNE (Figure 8c) were effectively diminished due to CHAC1 knockdown in mouse kidney stone models compared to the stone model group. Subsequently, we checked the expression of ferroptosis‐related proteins in the renal tissue using western blotting. The knockdown of CHAC1 significantly reversed the GPX4 and XCT protein deficiency in the mouse kidney stone model, and the upregulation of ACSL4 and CD71 protein was also mildly regained (Figure 8d,e). Simultaneously, the immunofluorescence analysis also demonstrated that CHAC1 deficiency ameliorated the GPX4 depletion (Figure 8f,h) and upregulated ACSL4 expression (Figure 8g,i) observed in the stone model group.

Additionally, we observed that in stone model AAV‐CHAC1 group, the damaged mitochondria, manifested by the reduction of mitochondrial cristae and the rupture of mitochondrial membrane, was apparently decreased compared to the stone model group (Figure 8j). Most importantly, the results of Von Kossa staining demonstrated that in the stone model+sh‐CHAC1 group, the area of crystal deposition was substantially decreased compared with the stone model group (Figure 8k). At the same time, the cell‐crystal adhesion proteins, CD44 and ANXA2, were significantly downregulated benefitting from the knockdown of CHAC1 in the stone model+AAV‐CHAC1 group (Figure 8l,m). Overall, these data that CHAC1 deficiency in kidneys could hinder the process of CaOx stone formation via inhibiting ferroptosis and ameliorating the renal impairment.

## 3 Discussion

Kidney stone, medically known as nephrolithiasis, is a common urological condition that has been affecting humans for centuries.^[^ ^28^ ^]^ Nephrolithiasis is prevalent worldwide, with a noticeable increase in incidence over the past few decades, which can cause severe pain, often described as one of the most intense types of pain a person can experience.^[^ ^3^ ^]^ Among all types of kidney stones, CaOx kidney stones accounts for more than 80% of them, thus exploring the mechanism of CaOx kidney stone formation is essentially needed.^[^ ^2^ ^]^ Currently, many scholars proposed that CaOx kidney stone formation is a step‐wise process from point to surface, namely, hyperoxaluria or CaOx crystals induce damage and death of local RTECs, and gradually cause damage to surrounding normal cells and tissues, thereby mediating crystal adhesion, aggregation and growth, and ultimately lead to the formation of CaOx kidney stone.^[^ ^7^, ^29^ ^]^ Therefore, unraveling the pathogenesis of RTEC injury in its infancy is particularly crucial for developing strategies to reduce the incidence and impact of kidney stones on individuals and healthcare systems.

**Figure 9:**
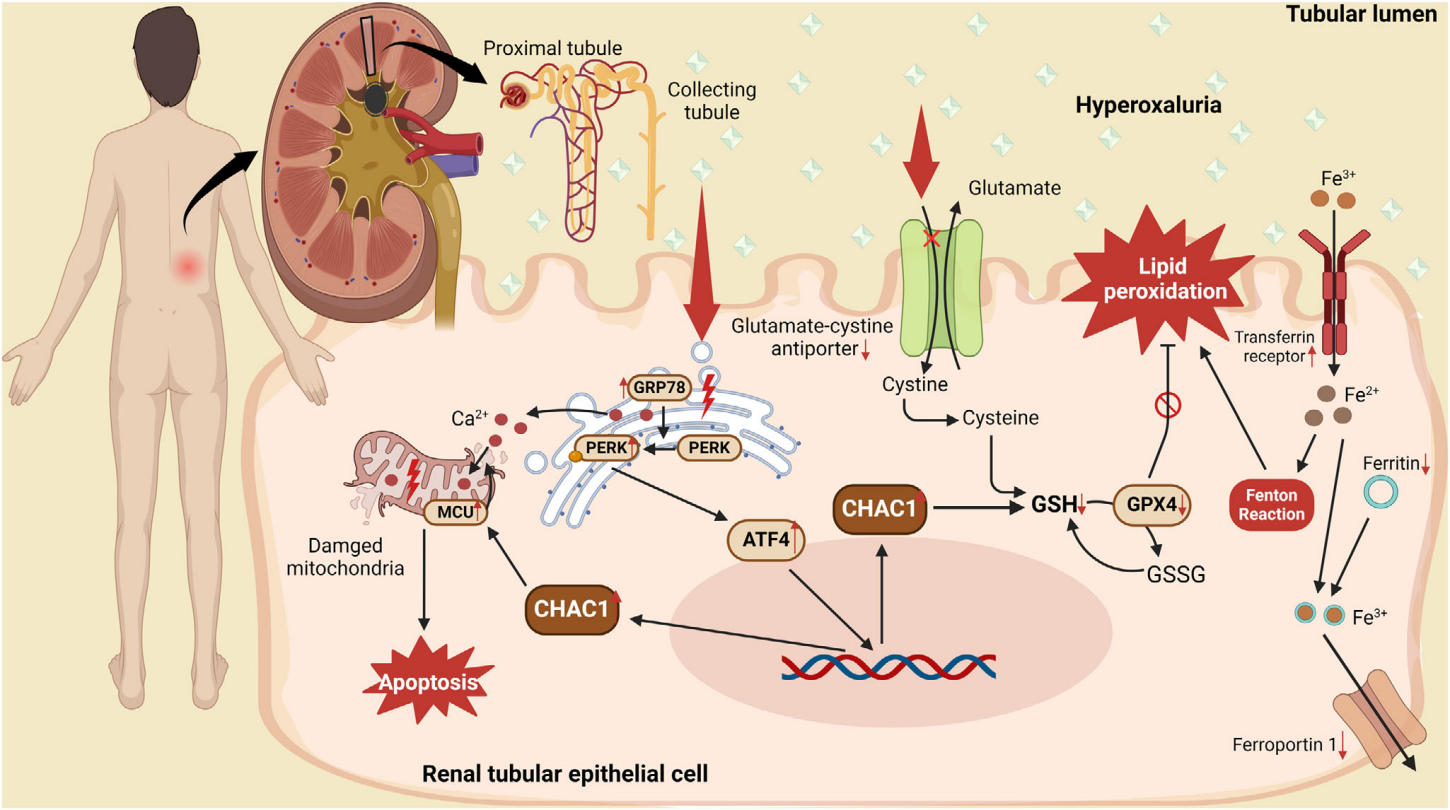
A schematic diagram illustrates the mechanism that CHAC1 mediates ER stress‐dependent ferroptosis in CaOx kidney stone formation.

In order to search for crucial clues about the mechanisms of RTEC injury stimulated by hyperoxaluria, we performed the 4D‐LFQ quantitative proteomic analysis, which clearly indicated that there existed ER stress‐related damage in vitro model. As we know, ER stress is a common cellular stress state often triggered by dysfunction or overload of the ER. When ER stress becomes excessive or prolonged, it can lead to violent inflammation response, apoptosis, autophagy, ferroptosis and so on.^[^ ^30^, ^31^, ^32^ ^]^ Our results confirmed the occurrence of excessive ER stress in vitro and in vivo stone models, and demonstrated the beneficial efficiency of regulating ER stress with 4‐PBA treatment for alleviating RTEC injury, improving renal impairment, and reducing crystal deposition in CaOx stone models. Simultaneously, the human transcription factors PCR Array plate was conducted to investigate the exact mechanism of ER stress‐mediated injury. Notably, ATF4 protein, also known as Activating Transcription Factor 4, stood out by this analysis. ATF4 was a central mediator of the stress response pathway, particularly in the UPR, which mediated cellular adaptations to stress and influenced various biological processes through the PERK/ATF4 pathway.^[^ ^33^, ^34^ ^]^ Subsequently, the downstream target genes of ATF4 were deeply explored using ChIP‐seq analysis. KEGG pathway annotation analysis of target genes suggested that the apoptosis and ferroptosis‐related genes were important downstream target of ATF4.

It has been reported that ER stress can induce oxidative stress, disturb calcium homeostasis, and trigger lipid peroxidation, which are all pivotal factors implicated in ferroptosis initiation.^[^ ^31^, ^35^ ^]^ Given our prior research findings that ferroptosis participated in the mechanism of RTEC injury,^[^ ^14^, ^22^ ^]^ we are the first to investigate the association of ER stress with ferroptosis in stone formation process. Specifically, ERS‐induced activation of UPR pathways, such as PERK and ATF6, can upregulate the expression of CHOP, a key mediator of ferroptosis.^[^ ^31^, ^36^ ^]^ Moreover, ER Stress‐mediated inhibition of cystine uptake through ablating XCT and the cystine/glutamate antiporter diminishes intracellular glutathione synthesis, thus reducing the capacity to neutralize lipid peroxides and sensitizing cells to ferroptosis.^[^ ^35^ ^]^ In our study, we found in Ox‐induced HK‐2 cell model that 4‐PBA treatment apparently alleviated the degree of ferroptosis, reducing MDA level, Fe^2+^ content and lipid peroxidation accumulation, as well as upregulating negative ferroptosis‐related proteins, including GPX4 and XCT. And additionally adding ER stress agonist further caused aggravation of ferroptosis level. Similarly, in mouse stone models, the degree of ferroptosis was attenuated after 4‐PBA treatment. These above results indicated that ER stress was involved in mediating the initiation of ferroptosis in CaOx kidney stone models.

Interestingly, in ChIP‐seq experiment, CHAC1 gene owned the highest fold enrichment in all ATF4‐target genes associated with UPR through GO annotation analysis. Similarly, based on the proteomics data and the nephrolithiasis gene expression dataset analysis, the gene expression correlation analysis indicated that CHAC1 may play an important role in mediating ER stress‐dependent ferroptosis in CaOx kidney stone formation. Furthermore, according to ChIP‐qPCR experiment, our study confirmed that there exists a significant interaction relationship between ATF4 protein and downstream CHAC1 gene. He and colleagues^[^ ^37^ ^]^ reported that in non‐alcoholic steatohepatitis (NASH)‐induced Hepatocellular carcinoma (HCC) models, ATF4 deficiency increased susceptibility to ferroptosis via decreasing XCT expression, leading to accelerated development of HCC. And several studies showed that inhibiting the activation of GRP78/PERK/ATF4 pathway restrained ER stress‐induced ferroptosis in multiple injury models.^[^ ^38^, ^39^, ^40^ ^]^Our study demonstrated that the PERK/ATF4/CHAC1 pathway was highly activated in CaOx stone models. Additionally, the ablation of ATF4 suppressed CHAC1 upregulation and pro‐ ferroptotic response triggered by Ox stimulation, which first suggested that ER stress‐dependent ferroptosis contributes to the molecular mechanism of CaOx stone formation via ATF4/CHAC1 pathway mediating.

Upregulation of CHAC1 expression is often as part of the cellular adaptive response.^[^ ^42^ ^]^ The molecular mechanism by which CHAC1 contributes to oxidative injury involves its enzymatic activity in degrading glutathione, a critical antioxidant involved in neutralizing ROS and protecting cells from oxidative damage.^[^ ^21^, ^43^ ^]^ This enzymatic reaction is facilitated by CHAC1's active site, which binds to glutathione and facilitates its cyclization, resulting in the formation of 5‐oxo‐L‐proline and Cys‐Gly dipeptide.^[^ ^44^ ^]^ We found the enrichment of CHAC1 and the depletion of GSH in both Ox‐stimulated HK‐2 cell model and glyoxylic acid‐induced mouse model. And CHAC1 deficiency alleviated oxalate‐induced inflammatory response and oxidative injury, and attenuated renal impairment and fibrosis in mouse stone models. Additionally, Mungrue and colleagues proposed that CHAC1 was a crucial downstream component of UPR, involving in proapoptotic pathway.^[^ ^17^ ^]^ And Cui and colleagues proposed that CHAC1 knockdown attenuated heat exposure‐induced IPEC‐J2 cell apoptosis.^[^ ^41^ ^]^ Our results suggested that knockdown of CHAC1 relieved the apoptotic status induced by Ox stimulation, and CHAC1 overexpression further worsen it. Based on RNA‐seq data, we found that, of all apoptosis‐related genes, MCU was most obviously downregulated after CHAC1 knockdown. MCU, mitochondrial calcium uniporter, is the core component of the MCU complex, which act as gatekeepers to maintain mitochondrial calcium homeostasis based on cytosolic calcium levels.^[^ ^42^ ^]^ Wherein, the regulation of calcium ion concentration between ER and mitochondria is vital for the appearance of apoptosis. Excessive calcium release from the ER can overwhelm mitochondrial calcium handling, resulting in mitochondrial dysfunction and triggering apoptotic pathways.^[^ ^43^, ^44^ ^]^ We found that CHAC1 deficiency caused the decrease of MCU protein expression, and the level of mitochondrial calcium ions was apparently decreased at the same time. And CHAC1 overexpression caused completely opposite effects on them. These indicated that CHAC1 deficiency may regulate MCU abundance to maintain the balance of mitochondrial calcium ions which in turn interrupt the apoptotic cascade at multiple levels, to a degree.

Most importantly, our results of RNA sequence experiment presented that CHAC1 also modulated ferroptosis‐related pathways. It is known that glutathione depletion is a key event in ferroptosis initiation. Wherein, the relationship between GPX4 and GSH is reciprocal and interdependent, and the enzymatic activity of GPX4 relies on the availability of GSH.^[^ ^45^ ^]^ GPX4 is an essential selenoprotein that belongs to the family of glutathione peroxidases, which plays a pivotal role in protecting cells against oxidative damage by catalyzing the reduction of lipid hydroperoxides to their corresponding alcohols, utilizing GSH as a cofactor.^[^ ^46^, ^47^ ^]^ Our current study demonstrated that CHAC1 deficiency significantly elevated the GSH level and GPX4 abundance in in vitro and in vivo CaOx kidney stone models, which further leads to reducing the products of lipid peroxidation and Fe^2+^ content. At the same time, the enrichment of XCT was also restored after CHAC1 knockdown. XCT facilitates the uptake of cystine, a precursor for producing intracellular cysteine, which serves as one of the three amino acids required for GSH synthesis.^[^ ^47^ ^]^ Thus, these results indicated that, in CaOx stone models, the simultaneous depletion of GSH due to its consumption and impaired biosynthetic sources can lead to GSH exhaustion, thereby triggering the onset of ferroptosis. Furthermore, CHAC1 overexpression partially eliminated the protective efficacy of 4‐PBA treatment on attenuating ferroptosis in Ox‐stimulated HK‐2 cells, and CHAC1 deficiency did cause positive regulation on ferroptosis in HK‐2 cells simultaneously stimulated by Ox and Tunicamycin, which further indicated that CHAC1 may be an important linker connecting ER stress with ferroptosis.

Based on above results, ferroptosis and apoptosis both appeared in CaOx kidney stone models. And CHAC1 deficiency simultaneously attenuated the status of both ferroptosis and apoptosis, to a certain extent. Recent studies established a continuous phenotypic gradient between ferroptosis and apoptosis, suggesting that these two pathways may not be entirely distinct but rather interconnected in their execution and regulation.^[^ ^48^ ^]^ While ferroptosis and apoptosis are distinct forms of cell death, they are interconnected through their responses to oxidative injury and their roles in inflammation.^[^ ^49^ ^]^ For instance, Vinik and colleagues reported that enhanced ER stress and alterations in phosphatidylethanolamine (PE) to phosphatidylcholine (PC) metabolism were implicated in the association between these two.^[^ ^48^ ^]^ In our study, the ferroptosis inhibitor, Ferrostatin‐1, significantly relieved the apoptotic condition in stone models. And the status of ferroptosis was also partially attenuated by Z‐VAD‐FMK alone treatment. Notably, the Ferrostatin‐1 and Z‐VAD‐FMK combination treatment better alleviating cell‐crystal adhesion and crystal composition than a single agent. And our current study demonstrated the cell‐crystal adhesion and crystal deposition were obviously improved as well due to CHAC1 deficiency. These results indicated that CHAC1 deficiency could effectively hinder CaOx kidney stone formation via simultaneously inhibiting ferroptosis and apoptosis. However, since these agent treatment and CHAC1 deficiency were not specific only to RTECs, but also acted on other cells in the renal, possibly including endothelial cells, mesangial cells or macrophages, and so on. Therefore, our present study only explains to a limited extent the potential effectiveness of blocking CaOx stone formation by inhibiting ER stress‐dependent ferroptosis and CHAC1 ablation.

## 4 Conclusion

We demonstrate, for the first time, that ER stress‐dependent ferroptosis is involved in the pathogenesis of CaOx kidney stones. And the molecular mechanism of ER stress‐dependent ferroptosis in CaOx stone formation may be caused by the activation of ATF4/CHAC1 pathway, and then the specific degradation of GSH by CHAC1 disrupts the production and biological activity of GPX4, which ultimately leads to ferroptosis. Besides, simultaneous inhibition of apoptosis and ferroptosis may enhance the prevention of stone formation, and CHAC1 deficiency has been shown to suppress both apoptotic and ferroptotic pathways (Figure 9). Based on our above findings, CHAC1 may represent a promising new therapeutic target for preventing or treating CaOx kidney stones.

## 5 Experimental Section

### Animal Experiments

The protocols for the animal experiments were strictly reviewed and approved by Laboratory Animal Welfare and Ethics Committee of Renmin Hospital of Wuhan University (Issue No. 20230705F). The study utilized a total of 78 six‐week‐old male KM mice, all of which were provided with standard food and water throughout the experimental period. These animal experiments were conducted in three parts, and each group in each part comprised six mice (*n* = 6 in each group). The experimental groups were structured as follows:

Part I: *Control group*: Mice received daily intraperitoneal injections of 0.1 ml normal saline for 14 days; *the 4‐Phenylbutyric acid (4‐PBA) control group*: Mice were administered daily intraperitoneal injections of 0.1 ml 4‐PBA (20  mg kg^−1^) (1821‐12‐1, MCE, USA) for 14 days; *the stone model group*: Mice were injected intraperitoneally with 0.1 ml glyoxylic acid (60 mg kg^−1^) (260150‐250G‐A, Sigma‐Aldrich, USA) daily for 14 days to establish the model; *the 4‐PBA treatment group*: Mice received daily intraperitoneal injections of a 0.1 ml mixture containing glyoxylic acid (60 mg kg^−1^) and 4‐PBA (20 mg kg^−1^) for 14 days.
Part II: The shRNA‐CHAC1 targeting mouse CHAC1 and negative control were individually encapsulated into the adeno‐associated virus (AAV) serotype 9 vectors, prepared by Obio Technology Corp., Ltd. (Shanghai, China). AAV vectors carrying either the negative control or shRNA‐CHAC1 (200µl, 5 × 10^11^vg) were administered via tail vein injection. The expression of CHAC1 was assessed after 4 weeks. *Control + AAV‐NC group*: four weeks after the administration of AAV containing negative control, mice received daily intraperitoneal injections of 0.1 ml normal saline for 14 days; *Control + AAV‐CHAC1 group*: four weeks following the administration of AAV carrying shRNA‐CHAC1, mice received daily intraperitoneal injections of 0.1 ml normal saline for 14 days; *Stone model + AAV‐NC group*: four weeks after the administration of AAV containing negative control, mice received intraperitoneal injections of 0.1 mL glyoxylic acid (60 mg kg^−1^) daily for 14 days; *Stone model + AAV‐CHAC1 group*: four weeks after the administration of AAV carrying shRNA‐CHAC1, mice received intraperitoneal injections of 0.1 mL glyoxylic acid (60 mg kg^−1^) daily for 14 days.
Part III: *Control group*: Mice received daily intraperitoneal injections of 0.1 ml normal saline for 14 days. *the stone model group*: Mice were injected intraperitoneally with 0.1 ml glyoxylic acid (60 mg kg^−1^) daily for 14 days. *the Ferrostatin‐1 treatment group*: Mice were injected intraperitoneally with 0.1 ml mixture of glyoxylic acid (60 mg kg^−1^) and Fer‐1 (5 mg kg^−1^) (HY‐100579, MCE, USA) for 14 days. *the Z‐VAD‐FMK treatment group*: Mice were injected intraperitoneally with 0.1 ml mixture of glyoxylic acid (60 mg kg^−1^) and Z‐VAD‐FMK (10 mg kg^−1^) (HY‐16658B, MCE, USA) for 14 days. *the Ferrostatin‐1 and Z‐VAD‐FMK combination treatment group*: Mice were injected intraperitoneally with 0.1 ml mixture of glyoxylic acid (60 mg kg^−1^), Fer‐1 (5 mg kg^−1^), and Z‐VAD‐FMK (10 mg kg^−1^) for 14 days.

After treatment for 14 days, urine and blood samples were collected from each mouse. Subsequently, all mice were anesthetized an euthanized, and their kidney tissues were harvested and preserved for subsequent analyses.

### Cell Culture and Intervention

Human proximal tubular epithelial cells (HK‐2), obtained from Procell Life Science & Technology Co., Ltd. (Wuhan, China), were utilized in these experiments. These cells were maintained in DMEM/F12 medium (Gibco, USA), supplemented with 1% penicillin‐streptomycin (C0222, Beyotime Biotech Inc, China) and 10% fetal bovine serum (FBS, Biological Industries, Israel). The culture conditions included a humidified incubator set to 37 °C with a 5% CO_2_ atmosphere. The medium was renewed daily, and subculturing was performed when the cell population reached 80–90% confluency. To establish a model of oxalate‐induced cell injury, oxalate solution (75 688, Sigma, USA) was employed.

### Cell Transfection and Interference

Lentiviral (LV) vectors for CHAC1 and ATF4 knockdown, along with the corresponding negative controls, were generated by Shanghai Genechem Co., Ltd. (Shanghai, China). When the HK‐2 cells reached 20% confluency, they were transfected with LV containing sh‐CHAC1 or sh‐ATF4 with assistance of transfection reagent. Stably transfected cell lines were then selected using the medium containing 2 µg ml^−1^ puromycin over a period of 7 days. Additionally, the plasmids for CHAC1 overexpression, along with their negative controls, were constructed by Tsingke Biotechnology Co., Ltd. (Beijing, China). Transfections were performed using CHAC1 plasmids with the help of Lipo2000 transfection reagent (11 668 019, Thermo Fisher Scientifc Inc., USA). The resulting stably transfected cell lines and transfected cells were subsequently utilized in further experiments. Detailed sequence information is provided in Table S1 (Supporting Information).

### 4D‐Label Free Quantitation (4D‐LFQ) Proteomic Analysis

HK‐2 cells were categorized into a normal group and a stone model group. Following the respective interventions, the culture medium was removed, and cell samples were harvested and stored at ‐80 °C. Protein concentrations were determined using the BCA assay (P0010, Beyotime, China). All samples underwent enzymatic digestion prior to analysis. Protein abundance was evaluated using liquid chromatography‐mass spectrometry (LC‐MS), and the resulting MS/MS data were processed using Maxquant search engine (v.1.6.15.0). Identified and quantified proteins in this study were analyzed based on their functions and characteristics. Subcellular localization was predicted using WoLF PSORT, while Gene ontology (GO) annotations were retrieved from the UniProt‐GOA database (http://www.ebi.ac.uk/GOA/). Additionally, Kyoto encyclopedia of genes and genomes (KEGG) pathway analysis was also performed to investigate differentially expressed proteins (DEPs).

### Transcription Factors PCR Array

The human Transcription Factors PCR Array plate (Wcgene Biotech, China) was employed to examine gene expression profiles strictly in accordance with the manufacturer's protocol. The raw data were processed using Wcgene Biotech's proprietary software. And genes with fold changes exceeding 2.0 or falling below 0.5 were considered to have biological significance.

### Bioinformatic Analysis

The nephrolithiasis gene expression dataset was obtained from GSE73680 (https://www.ncbi.nlm.nih.gov/geo/query/acc.cgi?acc=GSE73680) and then chosen for this investigation, which was consisted of 62 renal papillae tissues from 33 individuals without nephrolithiasis and 29 patients with nephrolithiasis. The correlation between GPX4 and CHAC1 was performed using this dataset.

### RNA Sequencing

After the respective interventions, total RNA was extracted with TRIzol Reagent (15 596 026, Invitrogen, USA). Then, RNA quality was assessed and quantified with Qubit3.0 fluorometer using the Qubit RNA Broad Range Assay kit (Q10210, Life Technologies, USA). Stranded RNA sequencing libraries were constructed using 2 µg of total RNA and the KC‐Digital Stranded mRNA Library Prep Kit for Illumina (DR08502, Wuhan Seqhealth, China) in accordance with the manufacturer's protocol. Trimmomatic (version 0.36) was utilized to filter and clean the raw sequencing data. Then, differentially expressed genes (DEGs) between Ox group and Ox+sh‐CHAC1 group were identified using the edgeR package (version 3.12.1) with a threshold of |_log_foldchange| ≥ 0.58. DEGs were analyzed for GO and KEGG enrichment using KOBAS software (version: 2.1.1), applying a P‐value threshold of 0.05 to define statistically significant enrichment.

### ChIP Sequencing

First, 1% formaldehyde was used to fix the HK‐2 cells at room temperature for 10 min, and then adding 0.125 m glycine for incubating 5 min to stop the cross‐linking reaction. HK‐2 cells were lysed using cell lysis buffer, and nuclei were separated by centrifugation at 2000 g for 5 min. Subsequently, the nucleus underwent treatment with lysis buffer and sonication to fragment chromatin DNA. The chromatin was processed as follows: 10% was reserved as “input”, 80% was subjected to immunoprecipitation with anti‐ATF4 antibody (10835‐1‐AP, Proteintech, USA) and labeled “IP”, while the remaining 10% was treated with rabbit IgG (Cell Signaling Technology) as a negative control, labeled “IgG”. DNA from both the input and IP samples was isolated using the phenol‐chloroform extraction method. DNA sequencing libraries were generated using the VAHTS Universal DNA Library Prep Kit for Illumina V3 (ND607, Vazyme, China). Library fragments ranging from 200 to 500 bps were enriched, quantified, and then sequenced using DNBSEQ‐T7 sequencer (MGI Tech Co., Ltd. China) with the PE150 configuration. Reads distribution analysis was conducted using the RSeQC (version 2.6). Peak calling was conducted with MACS2 software (Version 2.1.1). Peak distribution and annotation analysis were carried out using Bedtools (version 2.25.0). Subsequently, GO analysis and KEGG enrichment analyses were conducted using KOBAS software (version 2.1.1)

### ChIP qPCR

Based on the ChIP‐seq experiment, the distribution of ATF4 reads over the CHAC1 gene was validated. The predicted binding site of ATF4 protein targeting CHAC1 transcription were presented and the primers were designed in Table S2 (Supporting Information). In accordance with ChIP‐seq experimental protocols, DNA was extracted from both input and IP samples. Subsequently, Hieff UNICON Universal Blue qPCR SYBR Green Master Mix (11184ES08, Yeasen, China) was used to conduct qRT‐PCR on the LightCycler480 (Roche Diagnostics, USA). Raw data were calculated as percent input.

### Cell Viability Assay

HK‐2 cells were cultured in 96‐well plates until reaching 70% confluency. Following the corresponding intervention, the medium was discarded, and 100 µl of the CCK8 working solution (BS350B, Biosharp, China) was added to each well, followed by incubation for 2 h at 37 °C. Absorbance at 450 nm was recorded using a microplate reader (EnSight, PerkinElmer, USA).

### Cell Biochemistry Assay

The levels of T‐AOC, LDH, SOD, MDA, GSH, and Caspase 3 activity were measured using T‐AOC assay kit (A015‐2‐1, Nanjing Jiancheng, China), LDH assay kit (A020‐2‐2), SOD assay kit (A001‐3‐2), MDA assay kit (A003‐4‐1), GSH assay kit (A006‐2‐1), and Caspase 3 Activity Assay Kit (C1116) strictly following the manufacturers' instructions. Absorbance was determined using the microplate reader at the following wavelengths: 450 nm for LDH and SOD, 405 nm for T‐AOC, GSH, and Caspase 3, and 530 nm for MDA.

### Iron Content Analysis

The cellular Fe^2+^ level was visualized using FerroOrange assay (F374, DOJINDO, Japan). After the intervention, cells were incubated with 1 µm FerroOrange at 37 °C for 30 min. Fluorescence intensity was promptly examined under an inverted fluorescence microscope (IX71, Olympus, Japan) at ×400 magnification. Fluorescence intensity was subsequently quantified with ImageJ software (ImageJ 1.51j8 version, USA). Additionally, intracellular iron levels, including total iron and Fe^2^⁺, were measured using the Iron Assay Kit (I291, DOJINDO, Japan) in accordance with the manufacturer's protocol. Absorbance at 593 nm was recorded using the microplate reader.

### Mitochondrial Membrane Potential Measurement

Following the manufacturer's protocol, 2 ml of JC‐1 working solution, comprising 1 ml JC‐1 stain (C2006, Beyotime, China) and 1 ml basal medium, was added and incubated in each well at 37 °C for 20 min. Following incubation, the working solution was discarded, and the cells were rinsed twice with cold 1× JC‐1 staining buffer. The cells were then promptly visualized under the inverted fluorescence microscope at ×400 magnification. Fluorescence intensity was quantified using ImageJ software (ImageJ 1.51j8 version, USA).

### Intracellular ROS Levels Detection

Following the intervention, the working solution containing 10 µmol l^−1^ DCFH‐DA (Beyotime, China) was performed to treat cells for 30 min. Intracellular ROS levels were measured using a flow cytometer (CytoFlex, Beckman Coulter, USA). FlowJo software (FlowJo 10.6.2 version, USA) was used to analyze the fluorescence intensity. Additionally, intracellular ROS levels were visualized under the inverted fluorescence microscope at ×200 magnification, and fluorescence intensity was analyzed with the ImageJ software.

### Lipid Peroxidation Level

Lipid peroxidation levels were evaluated using the Lipid Peroxidation Probe‐BDP 581/591 C11 (L267, Dojindo, Japan). Cells from all groups were incubated with the probe for 30 min at 37 °C. Fluorescence signals indicative of lipid peroxidation were visualized using the inverted fluorescence microscope at ×400 magnification and quantified with ImageJ software (version 1.51j8, USA). Otherwise, the level of lipid peroxidation was also measured using the Liperfluo assay (L248, Dojindo, Japan) in conjunction with the flow cytometer. The flow cytometry data were further visualized and analyzed using the FlowJo software.

### Measurement of Calcium Ion Levels

For detecting intracellular Ca^2+^ content, each group was incubated with the working solution containing 5 µm Fluo‐4, AM (40704ES50, Yeasen, China) and 0.05% F‐127 (60318ES60, Yeasen, China) for 40 min at 37 °C. For detecting mitochondrial Ca^2+^ content, each group was incubated with the working solution containing 5 µm Rhod‐2, AM (40776ES72, Yeasen, China) and 0.05% F‐127 (60318ES60, Yeasen, China) for 40 min at 37 °C. The cells were promptly examined using a confocal scanning laser microscopy (FV1200, Olympus, Japan) at ×1000 magnification, and fluorescence intensity was analyzed with the ImageJ software.

### Analysis of Apoptotic Cells

The apoptotic status was assessed using ANNEXIN V‐FITC/PI apoptosis detection kit (E‐CK‐A211, Elabscience) in accordance with the manufacturer's protocol. Cells from each sample were sorted using the flow cytometer, and the data were processed with the FlowJo software.

### Cell Adhesion Ability Analysis

Fibronectin (F8180, Solarbio) was pre‐coated onto 96‐well plates. Cells were then seeded onto the prepared plates and cultured for 4h. Subsequently, Phosphate buffer saline (PBS) washed the 96‐well plate three times to remove the unadhered cells. Finally, the CCK8 working solution (BS350B, Biosharp, China) was used to calculate the number of adhered cells. The cell adhesion ability was the ratio of adhered cells to the total number of seeded cells.

### Cell–Crystal Adhesion

Following the intervention, the waste medium was removed, and cell samples were washed five times with 1 ml of Hanks' Balanced Salt Solution (HBSS). During washing, the culture plate was agitated on a shaker at 150 rpm for 2 min. Ultimately, the cells were promptly examined using the inverted microscope at ×400 magnification, and the cell‐crystal adhesion area was measured with the ImageJ software.

### Quantitative Real‐Time Reverse Transcription PCR (qRT‐PCR)

Following cell intervention, total RNA was isolated from the treated cells using TRIzol reagent (15 596 026, ThermoFisher, USA) as per the manufacturer's instructions. Subsequently, 2 µl RNA was used for cDNA synthesis with the Hifair III 1st Strand cDNA Synthesis Kit (gDNA digester plus) (11139ES60, Yeasen, China). qRT‐PCR was set up on LightCycler480 (Roche Diagnostics, USA) with Hieff UNICON Universal Blue qPCR SYBR Green Master Mix (11184ES08, Yeasen, China). GAPDH served as the normalization reference, and relative expression changes were determined using 2^−△△Ct^ method. Details of primer sequences are presented in Table S3 (Supporting Information).

### Immunofluorescence Analysis

HK‐2 cells were cultured on slides and provided with corresponding treatment. Following the intervention, the medium was discarded and the cells were rinsed twice with PBS. They were then sequentially treated with 4% paraformaldehyde (P0099, Beyotime, China) for 15 min, 0.1% Triton X‐100 (T8200, Solarbio, China) for 10 min, and 1% BSA (ST2249, Beyotime, China) at room temperature for 1 h. Primary antibodies targeting CHAC1, GPX4, and XCT (detailed in Table S4, Supporting Information) were applied, and the slides were incubated at 4 °C for 12 h. After washing for three times with PBS, the cells were incubated with the secondary antibodies at room temperature for 1 h, and the sections were sealed using the anti‐fluorescence quencher (P0131, Beyotime, China). Fluorescence intensity was visualized using a positive fluorescence microscope (BX53, Olympus, Japan) at ×400 magnification.

### Western Blotting

Following corresponding intervention, total proteins from cells and kidney tissues were fully extracted using cold RIPA buffer (G2002, Servicebio, China). Extracted protein samples were separated by electrophoresis, transferred to PVDF Membrane (1 620 177, BIO‐RAD, USA), blocked with rapid blocking buffer (PS108P, Epizyme, China) for 15 min, washed four times with TBST, and incubated with primary antibodies at 4 °C for 12 h. The primary antibodies included anti‐GRP78, anti‐PERK, anti‐P‐PERK, anti‐IRE, anti‐P‐IRE, anti‐ATF4, anti‐CHOP, anti‐CHAC1, anti‐GPX4, anti‐ACSL4, anti‐XCT, anti‐FTH1, anti‐CD71, anti‐CD44, anti‐ANXA2, anti‐Nrf2, anti‐NQO‐1, anti‐BAX, anti‐BCL2, anti‐IL‐18, anti‐IL‐1β, and anti‐MCU with detailed information provided in Table S4 (Supporting Information). Following primary antibody incubation, the membranes were washed four times with TBST and then incubated with secondary antibodies at room temperature for 1 h. Each protein relative expression level was visualized using an Odyssey dual color infrared laser imager (LI COR, USA) and a ChemiDoc XRS system (BIO‐RAD, USA), with gray value analysis conducted via ImageJ software (ImageJ 1.51j8 version, USA).

### Enzyme‐Linked Immunosorbent Assay (ELISA)

Kim‐1 and NGAL levels in mouse renal tissue were determined using ELISA kits (CSB‐E08809 m and CSB‐E09410 m, Cusabio, China). Samples, along with standards and reagents, were prepared and incubated strictly following the manufacturer's protocol. The microplate reader was employed to measure the OD value at 450 nm.

### Blood Urea Nitrogen (BUN) and Serum Creatinine (CRE)

Serum BUN and CRE levels were determined using the BUN assay kit (C013‐2‐1, njjcbio, China) and CRE assay kit (C011‐2‐1, njjcbio, China). Samples and standard‐reagent mixtures were prepared and incubated precisely according to the manufacturer's instructions. The microplate reader was employed to measure the OD value at 640 nm for BUN and 546 nm for CRE.

### Immunohistochemistry (IHC)

Kidney tissues were harvested, fixed in 4% paraformaldehyde and embedded in paraffin. The paraffin‐embedded tissue block was sectioned, treated with xylene for 30 min, and then dewaxed using an ethanol gradient solution. Following antigen repair, the sections were incubated with antibodies against GPX4, ATF4, CHAC1, ACSL4, CD44, ANXA2, NGAL, and MCU at 4 °C for 12 h (detailed antibody information is provided in Table S4, Supporting Information). Subsequently, secondary antibodies were applied to the sections and incubated at room temperature for 1 h. Following washing, staining, counterstaining, dehydration, and sealing, the tissue sections were examined using the positive fluorescence microscope.

### Immunohistofluorescence (IHF)

Kidney tissues were harvested, fixed in 4% paraformaldehyde and embedded in paraffin. The paraffin‐embedded tissue block was sectioned, treated with xylene for 30 min, and then dewaxed using an ethanol gradient solution. Following antigen repair, the sections were incubated with antibodies against GPX4, CHAC1, 4‐HNE, KIM‐1, and α‐SMA at 4 °C for 12 h (detailed antibody information is available in Table S4, Supporting Information). Secondary antibodies were applied to the sections and incubated at room temperature for 1 h. Following washing, staining, counterstaining, dehydration, and sealing, the tissue sections were examined with the positive fluorescence microscope.

### Von Kossa Staining and Masson Staining

Kidney samples were preserved using 10% paraformaldehyde and subsequently embedded in paraffin. Following sectioning and dewaxing, the tissue sections underwent counterstaining with Von Kossa staining and Masson staining. These stained tissue sections were then observed using the positive fluorescence microscope.

### Transmission Electron Microscopy

HK‐2 cells were gently scraped and collected at room temperature. The treated HK‐2 cells and fresh renal tissues were promptly fixed using electron microscope fixation liquid (G1102, Servicebio, China). Through a series of steps, the mitochondrial structure was then observed using a transmission electron microscope (TEM) (HT7800, Hitachi, Japan).

### TUNEL Staining

The apoptotic status of renal tissue was assessed using the TUNEL kit (C1086, Beyotime, China). Kidney samples were sectioned, dewaxed, and treated with proteinase K for 15 min. The tissue sections were treated with TUNEL working solution and incubated at 37 °C for 1 h in the dark and subsequently examined under the positive fluorescence microscope.

### Statistical Analysis

This data, presented as mean ± standard deviation, were all obtained from at least three independent experiments. Comparisons between two groups were performed using Student's t‐test, while differences among multiple groups were analyzed using one‐way analysis of variance (ANOVA) followed by Turkey's multiple‐comparison test. Statistical analyses were conducted using GraphPad Prism 10.3.1 (263) (GraphPad Software, USA). The P‐value threshold of 0.01 was applied to define statistically significant difference.

### Ethics Approval and Consent to Participate

The animal experiment protocols were approved by Laboratory Animal Welfare and Ethics Committee of Renmin Hospital of Wuhan University (Issue No. 20230705F).

## Conflict of Interest

The authors declare no conflict of interest.

## Supporting information



Supporting Information

Supplemental Table 1

Supplemental Table 2

Supplemental Table 3

Supplemental Table 4

## Data Availability

The data that support the findings of this study are available from the corresponding author upon reasonable request.
