# Supplementary material for: CHAC1 Mediates Endoplasmic Reticulum Stress‐Dependent Ferroptosis in Calcium Oxalate Kidney Stone Formation
Source: Adv Sci (Weinh). 2025 Jan 21;12(10):2403992. doi: 10.1002/advs.202403992 (PMC11905043; doi:10.1002/advs.202403992)
Supplement: Supplementary file 1 — Supporting Information [file ADVS-12-2403992-s002.docx]

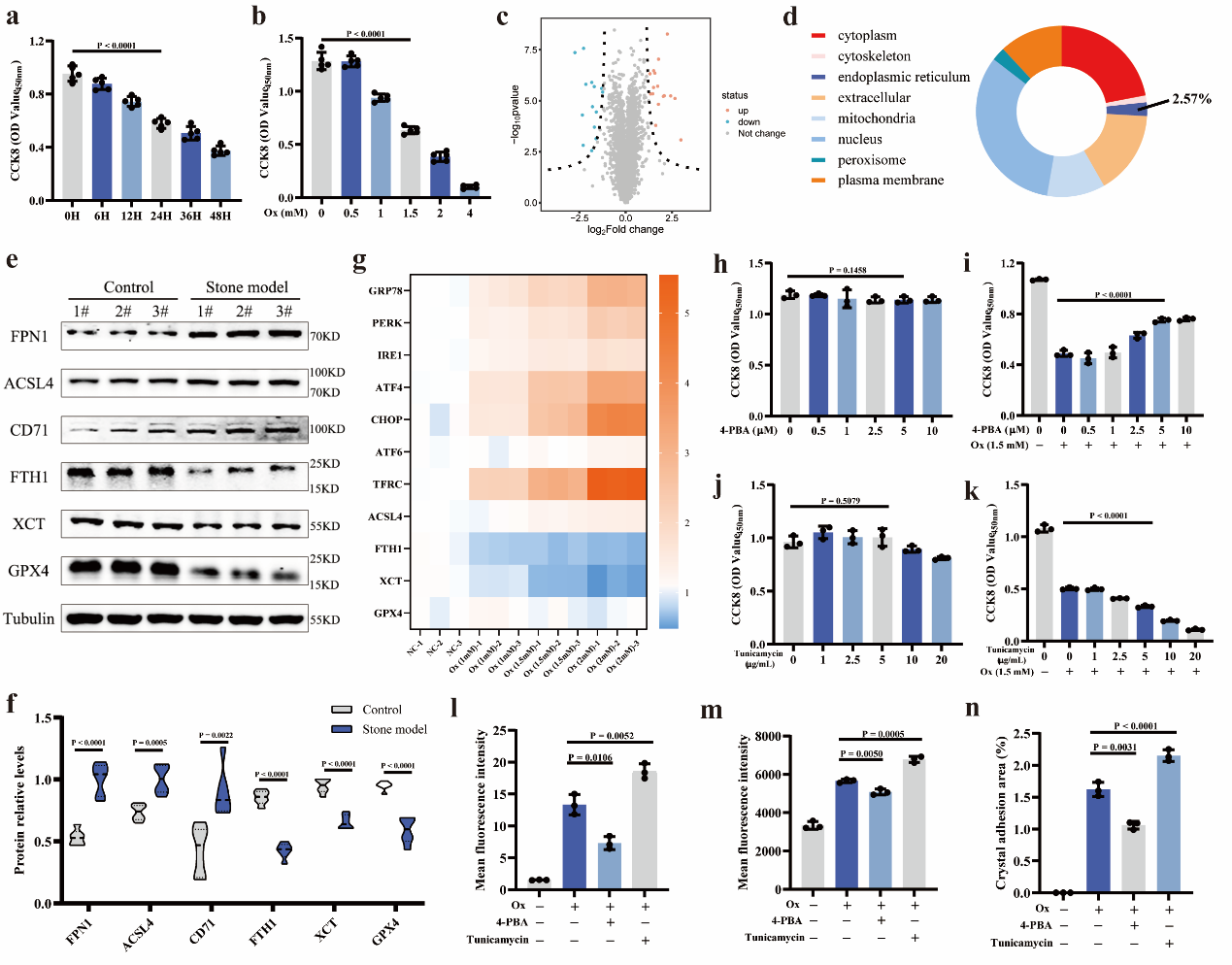


**Figure S1.** (a) HK-2 cells were incubated for time periods and CCK-8 assay was used to detect cell viability. (b) HK-2 cells were treated with varying concentrations of Ox and CCK-8 assay was used to detect cell viability. (c) Volcano plot for DEPs in 4D-Label Free Quantitation (4D-LFQ) proteomic analysis. (d) Subcellular location of DEPs in 4D-LFQ proteomic analysis. (e) Western blotting results showed the protein expression levels of FPN1, ACSL4, CD71, FTH1, XCT and GPX4 for stone model group and control group and (f) the violin plots show the protein relative expression. (g) qPCR results show the transcriptional levels of GRP78, PERK, IRE1, ATF4, CHOP, ATF6, CD71, ACSL4, FTH1, XCT and GPX4 followed by Ox concentration gradient incubating for 24h. (h) HK-2 cells were treated with different concentrations of 4-PBA for 24h and then the cell viability was determined. (i) HK-2 cells were treated with 1.5 mM oxalate in the presence of coincubation with different concentrations of 4-PBA for 24h and then the cell viability was determined. (j) HK-2 cells were treated with different concentrations of Tunicamycin for 24h and then the cell viability was determined. (k) HK-2 cells were treated with 1.5 mM oxalate in the presence of coincubation with different concentrations of Tunicamycin for 24h and then the cell viability was determined. (l) Bar graph shows cellular ROS levels. (m) Bar graph shows cellular lipid peroxidation levels. (n) Bar graph shows the positive area of crystal adhesion. Based on three independent experiments, data are presented as means ± SEM. P value is directly displayed and P values < 0.01 is considered significant.


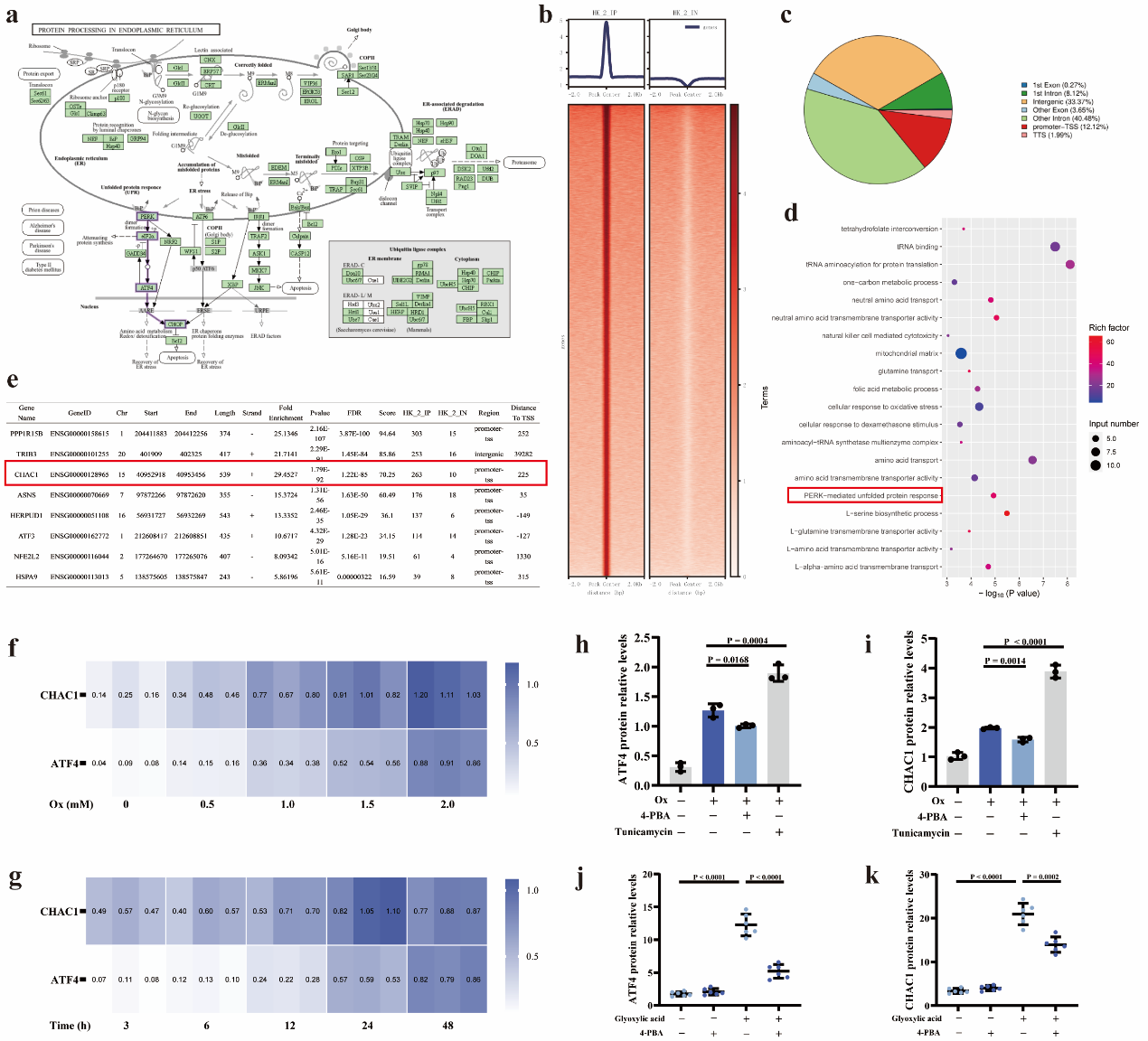


**Figure S2.** (a) The map of KEGG pathway termed protein processing in endoplasmic reticulum. (b) The distribution of reads on both sides of the peak center in ChIP-seq experiment. (c) Peak distribution in different genomic regions in ChIP-seq experiment. (d)Bubble plot for GO annotation analysis of target genes in ChIP-seq experiment. (e) Based on GO annotation analysis of target genes, all target genes associated with ER stress were screened. (f) Heatmap shows the protein relative expression levels of ATF4 and CHAC1 followed by Ox concentration gradient. (g) Heatmap results show the protein relative expression levels of ATF4 and CHAC1 followed by time gradient. (h) Bar graph shows the ATF4 protein relative expression. (i) Bar graph shows the CHAC1 protein relative expression. (j) Bar graph shows the ATF4 protein relative expression in renal tissues. (k) Bar graph shows the CHAC1 protein relative expression in renal tissues. Based on three independent experiments, data are presented as means ± SEM. P value is directly displayed and P value < 0.01 is considered significant.


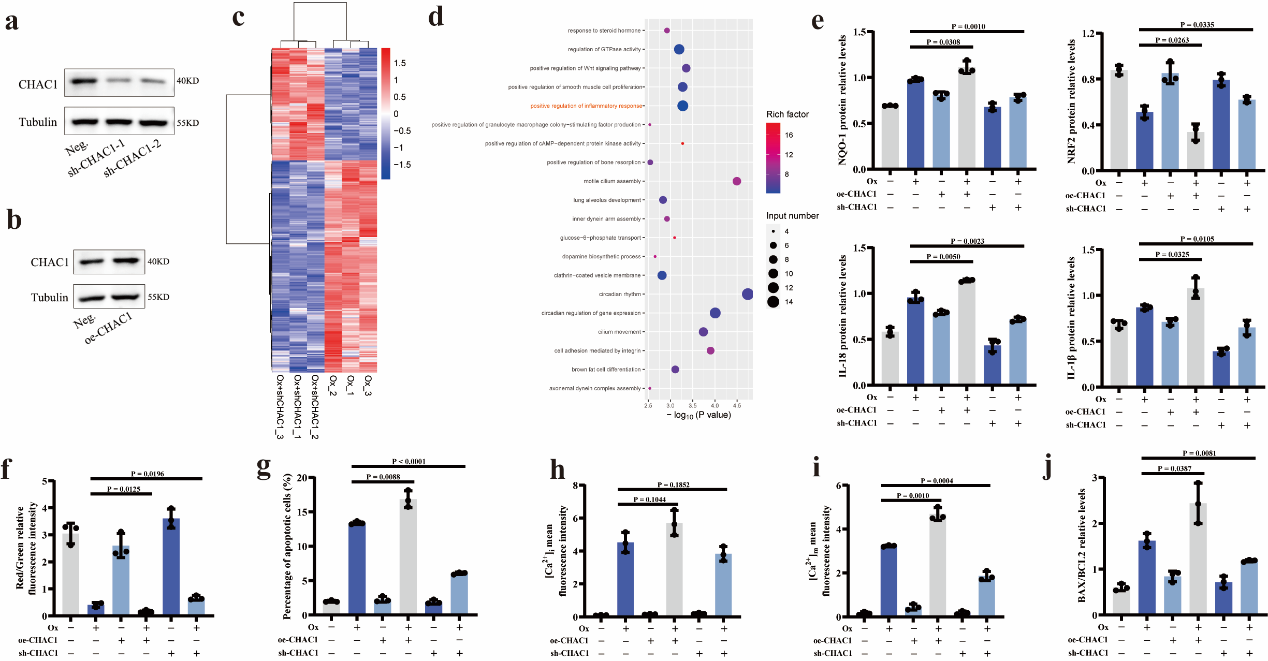


**Figure S3.** (a) Validation of CHAC1 knockdown by western blot. (b) Validation of CHAC1 overexpression by western blot. (c) Heatmap for DEPs in RNA-seq experiment. (d) Bubble plot for GO enrichment analysis in RNA-seq experiment. (e) Bar graphs show the NQO-1, Nrf2, IL-18, and IL-1β protein relative expression, respectively. (f) Bar graph shows the ratio of red to green fluorescence. The lower the ratio, the worse the degree of lipid peroxidation. (g) Bar graph shows percentage of apoptotic cells. (h) Bar graph shows the levels of intracellular Ca^2+^. (i) Bar graph shows the levels of mitochondrial Ca^2+^. (j) Bar graph shows the relative expression ratio of BAX to BCL2 protein. Based on three independent experiments, data are presented as means ± SEM. P value is directly displayed and P value < 0.01 is considered significant.


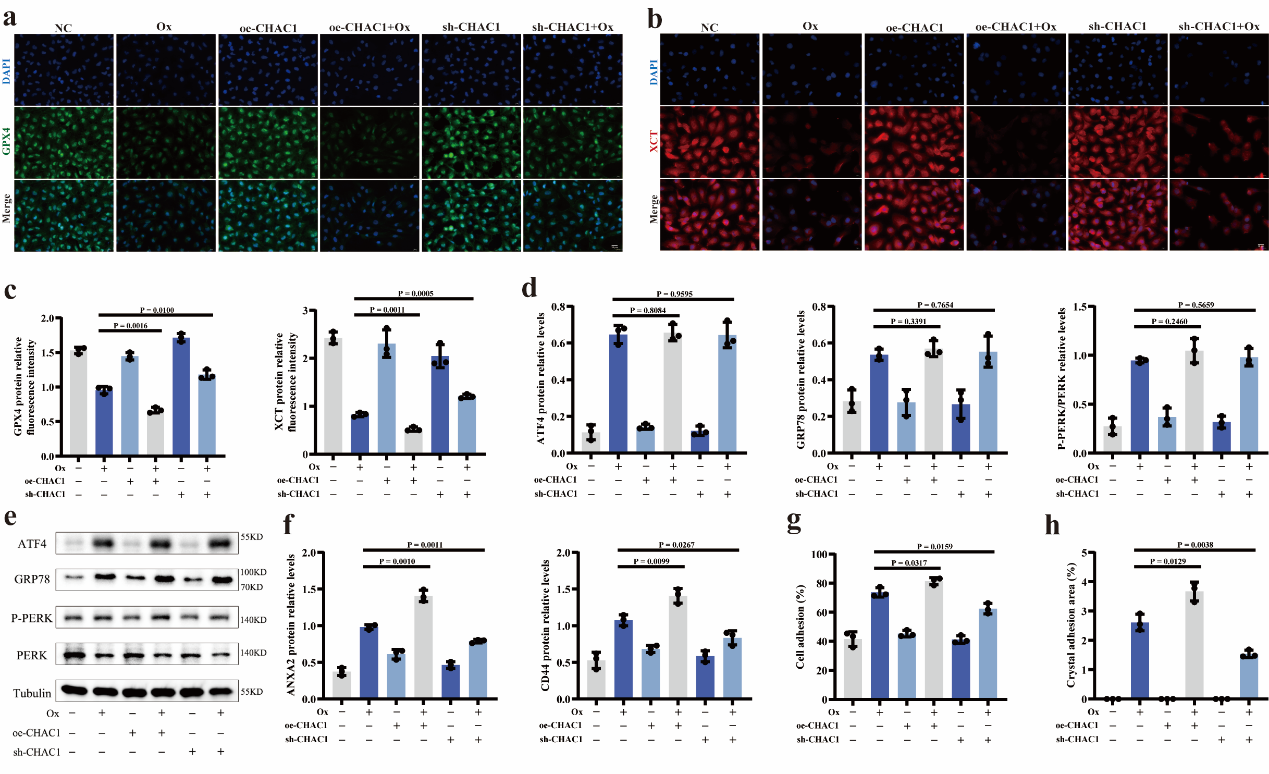


**Figure S4.** (a) Immunofluorescence staining was performed to detect GPX4 protein expression for six groups (magnification, ×400). (b) Immunofluorescence staining was performed to detect XCT protein expression for six groups (magnification, ×400). (c) Bar graphs show the GPX4 and XCT protein relative levels. (d) Bar graphs show the ATF4 and GRP78 protein relative levels and the expression ratio of P-PERK to PERK protein. (e) Western blotting results show the protein expression levels of ATF4, GRP78, P-PERK and PERK for six groups. (f) Bar graphs show the ANXA2 and CD44 protein relative levels. (g) The ability of cell adhesion was detected. (h) Bar graph shows the positive area of crystal adhesion. One set of representative images of three independent experiments is shown. Based on three independent experiments, data are presented as means ± SEM. P value is directly displayed and P value < 0.01 is considered significant.


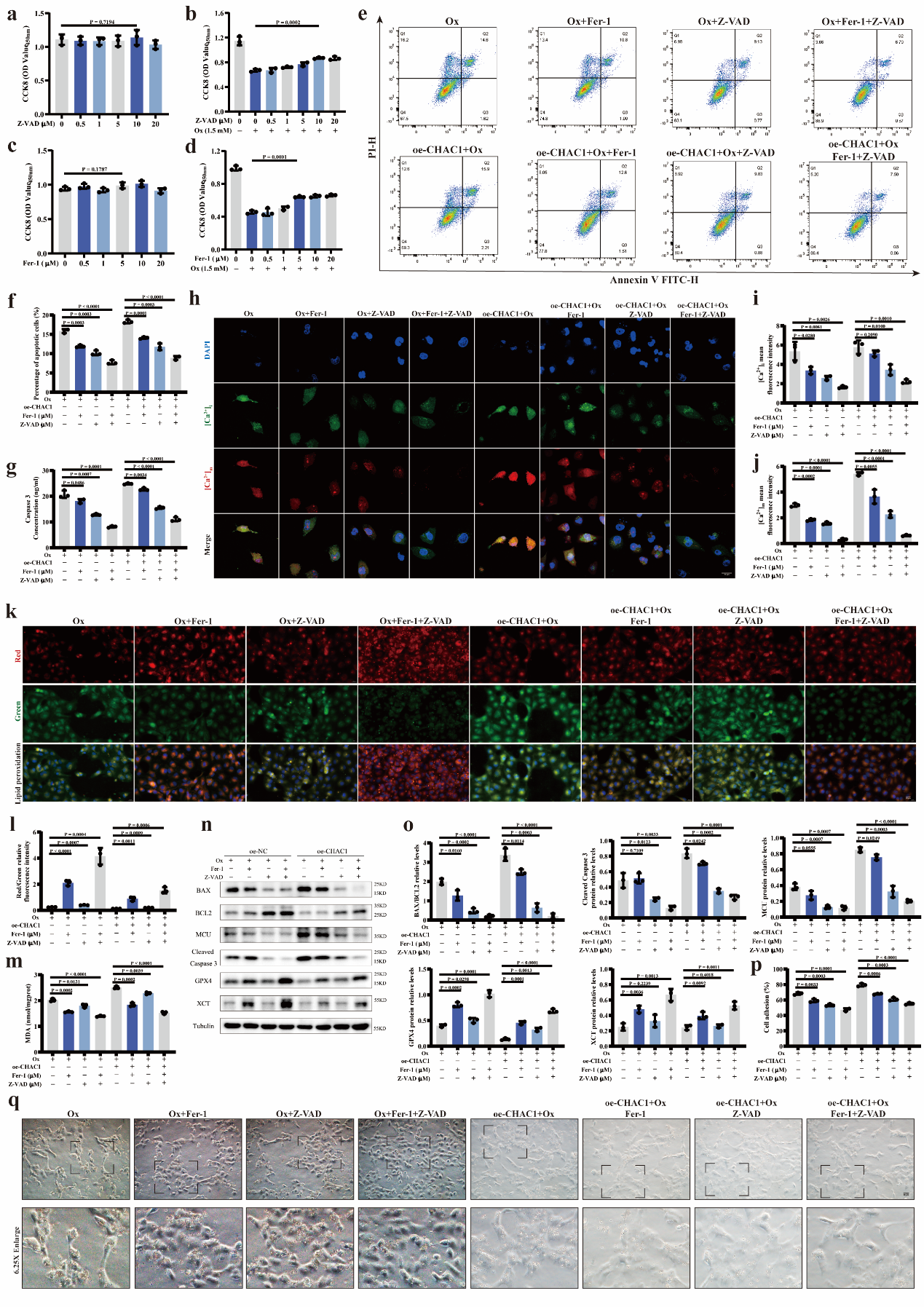


**Figure S5.** (a) HK-2 cells were treated with different concentrations of Z-VAD-FMK for 24h and then the cell viability was determined. (b) HK-2 cells were treated with 1.5 mM oxalate in the presence of coincubation with different concentrations of Z-VAD-FMK for 24h and then the cell viability was determined. (c) HK-2 cells were treated with different concentrations of Ferrostatin-1 for 24h and then the cell viability was determined. (d) HK-2 cells were treated with 1.5 mM oxalate in the presence of coincubation with different concentrations of Ferrostatin-1 for 24h and then the cell viability was determined. (e) The flow cytometry was used detect the apoptotic condition for eight groups and (f) bar graph shows percentage of apoptotic cells. (g) Bar graph shows the level of cellular caspase 3 activity. (h) The Fluo-4, AM and Rhod-2, AM were used to detect intracellular Ca^2+^ content and mitochondrial Ca^2+^ content, respectively (magnification, ×1000). (i) Bar graph shows the levels of intracellular Ca^2+^. (j) Bar graph shows the levels of mitochondrial Ca^2+^. (k) The BDP 581/591 C11 assay was used to qualitatively measure the degree of lipid peroxidation (magnification, ×400) and (l) bar graph shows the ratio of red to green fluorescence. The lower the ratio, the worse the degree of lipid peroxidation. (m) The MDA content was measured. (n) Western blotting results show the protein expression levels of BAX, BCL2, Cleaved Caspase 3, MCU, GPX4 and XCT for eight groups. (o) Bar graphs show the Cleaved Caspase 3, MCU, GPX4 and XCT protein relative levels and the expression ratio of BAX to BCL2 protein. (P) The ability of cell adhesion was detected. (q) Images show the status of cell-crystal adhesion between Ox crystals and HK-2 cells (magnification, ×400). One set of representative images of three independent experiments is shown. Based on three independent experiments, data are presented as means ± SEM. P value is directly displayed and P value < 0.01 is considered significant.


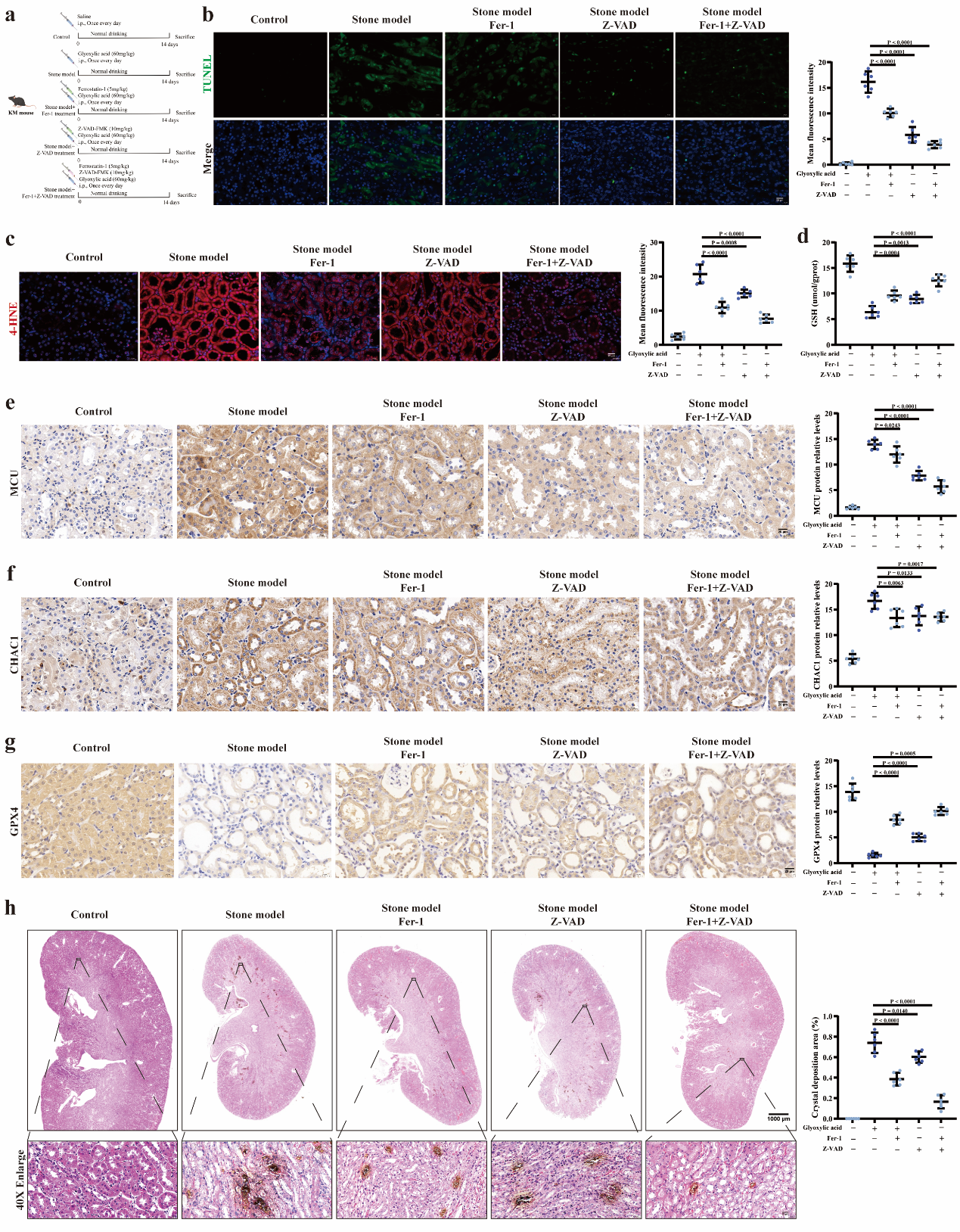


**Figure S6.** (a) A flowchart of the animal experiments. (b) The TUNEL staining was performed to detect the apoptotic level in renal tissue (magnification, ×400) and bar graph show the mean green fluorescence intensity. (c) Images show the 4-HNE expression abundance in renal tissue for four groups (magnification, ×400) and bar graph shows the mean red fluorescence intensity of 4-HNE expression. (d) The renal GSH content was measured. (e) Immunohistochemical staining was performed to measure the expression abundance of MCU protein and bar graph shows the protein relative expression (magnification, ×400). (f) Immunohistochemical staining was performed to measure the expression abundance of CHAC1 protein and bar graph shows the protein relative expression (magnification, ×400). (g) Immunohistochemical staining was performed to measure the expression abundance of GPX4 protein and bar graph shows the protein relative expression (magnification, ×400). (h) Von Kossa staining was used to display the status of crystal deposition in renal tissue (magnification, ×10 and ×400). Animal experiments were based on six independent experiments. One set of representative images of six independent experiments is shown. All data are presented as means ± SEM. P value is directly displayed and P value < 0.01 is considered significant.
